# Mapping tenascin-C interaction with toll-like receptor 4 reveals a new subset of endogenous inflammatory triggers

**DOI:** 10.1038/s41467-017-01718-7

**Published:** 2017-11-17

**Authors:** Lorena Zuliani-Alvarez, Anna M. Marzeda, Claire Deligne, Anja Schwenzer, Fiona E. McCann, Brian D. Marsden, Anna M. Piccinini, Kim S. Midwood

**Affiliations:** 1 Kennedy Institute of Rheumatology, Nuffield Department of Orthopaedics, Rheumatology and Musculoskeletal Sciences, University of Oxford, Roosevelt Drive, Headington, Oxford, OX3 7FY UK; 2 Structural Genomics Consortium, Nuffield Department of Clinical Medicine, University of Oxford, Roosevelt Drive, Headington, Oxford, OX3 7DQ UK; 3 Present Address: School of Pharmacy, University of Nottingham, Nottingham, NG7 2RD UK

## Abstract

Pattern recognition underpins innate immunity; the accurate identification of danger, including infection, injury, or tumor, is key to an appropriately targeted immune response. Pathogen detection is increasingly well defined mechanistically, but the discrimination of endogenous inflammatory triggers remains unclear. Tenascin-C, a matrix protein induced upon tissue damage and expressed by tumors, activates toll-like receptor 4 (TLR4)-mediated sterile inflammation. Here we map three sites within tenascin-C that directly and cooperatively interact with TLR4. We also identify a conserved inflammatory epitope in related proteins from diverse families, and demonstrate that its presence targets molecules for TLR detection, while its absence enables escape of innate immune surveillance. These data reveal a unique molecular code that defines endogenous proteins as inflammatory stimuli by marking them for recognition by TLRs.

## Abstract (web summary)

Although detection of pathogens by pattern recognition receptors is increasingly well defined, recognition of endogenous triggers remains poorly understood. By examining the interface between tenascin-C and TLR4, the authors identify a molecular code that identifies endogenous proteins as inflammatory stimuli.

## Introduction

Detailed insights into the molecular basis of pathogen detection by pattern recognition receptors (PRRs) have revealed how the toll-like receptor (TLR) family sense ligands including bacterial lipopolysaccharide (LPS), fungal glycans, protozoan DNA, and viral RNA (reviewed in refs. ^1–3^), as well as the way in which nod-like receptors recognize bacterial invasion^4, 5^ and rice pathogens^6^. These data demonstrate how a specific immune response to distinct microbial threat is mediated, enabling rational drug design to combat infectious disease. However, immunity has evolved to protect us from threat beyond pathogen invasion, and additional inflammatory triggers exist. Endogenous molecules specifically expressed, released, or secreted upon tissue injury are detected by the same PRRs that sense pathogenic ligands. These damage-associated ligands mediate tissue repair upon sterile injury but also contribute significantly to chronic inflammation associated with autoimmune, fibrotic, and metabolic diseases, and many are associated with tumorigenesis (reviewed in refs. ^7, 8^). The identification of endogenous triggers of inflammation raises the question of how PRRs detect this specific subset of molecules, while the majority of our self remains immunologically silent.

TLR4 is a relatively promiscuous immune sensor that recognizes both microbial and endogenous ligands, as well as allergens. Activation of TLR4 by pathogenic stimuli has been extensively studied^9–11^, and details of its activation by the metal allergen nickel^12^ and the house dust mite allergen Der p2^13^ are emerging. However, it is not clear how TLR4 detects endogenous inflammatory stimuli; no structural data are available, and it is poorly understood on the molecular level what, specifically, about these agonists enables their recognition by TLR4, while other molecules escape the attention of this PRR. Tenascin-C is an extracellular matrix protein that exhibits a restricted pattern of expression in healthy tissues, but which is specifically upregulated at sites of tissue injury^14^, where it triggers inflammation by activating TLR4 in cells including macrophages, dendritic cells, neutrophils, chondrocytes, and synovial fibroblasts^15–17^. Tenascin-C has been implicated as one of the major driving factors of chronic inflammation in diseases including rheumatoid arthritis^15, 18^, asthma^19^, tumors^20^, and fibrosis^21^. However, lack of information about the mode of TLR4 activation by tenascin-C has prevented the design of specific, effective antagonists.

Here we investigate how tenascin-C is detected by TLR4. Our data pinpoint three specific sites within this large, multi-domain matrix molecule that work together to interact with and activate TLR4. We also demonstrate that this inflammatory epitope exists in other molecules from distinct protein families that contain homologous domains, where it defines TLR4 recognition. Together these data begin to unravel the molecular basis of endogenously driven immunity, revealing a common code that earmarks “dangerous” proteins for detection by innate immune sensors, and which provides a tractable target for specifically reducing pathological “sterile” inflammation.

## Results

### Analysis of TLR activation by tenascin family members

**Fig. 1:**
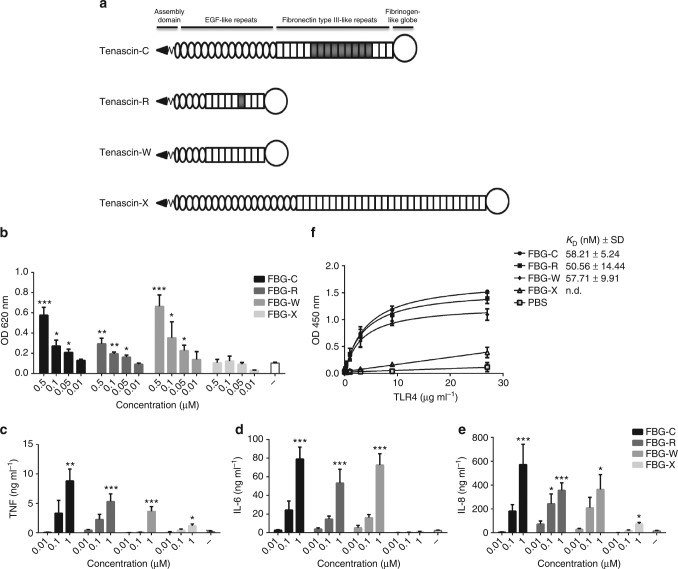
The FBG domains of tenascin-C, -R, and -W can induce NF-kB activation and cytokine synthesis, and bind to TLR4. **a** Tenascin-C, -R, -W, and -X each contain an assembly domain, a variable number of epidermal growth factor (EGF)-like repeats, a variable number of fibronectin type III-like repeats (these can be constitutively expressed (white rectangles) or alternatively spliced (gray rectangles) and a C-terminal fibrinogen-like globe (FBG) domain. The FBG domains exhibit a similar molecular weight, comprising between 229 and 240 amino acids each (FBG-C: 26.1 kDa, amino acids 1974–2201, FBG-R: 27.0 kDa, amino acids 1128–1359, FBG-W: 27.5 kDa, amino acids 1060–1300, FBG-X: 26.1 kDa, amino acids 4013–4243); protein accession numbers: tenascin-C (P24821), tenascin-R (Q92752), tenascin-W (Q9UQP3), tenascin-X (P22105). **b** THP1 NF-kB cells were stimulated with different concentrations of FBG-C, -R, -W, and -X, or were left unstimulated (−) for 24 h and NF-kB activation measured using QUANTI-Blue. Data are shown as mean ± SEM from three independent experiments. One-way ANOVA vs. non-stimulated, **p < 0.01, ***p < 0.001. **c**–**e** Primary human macrophages were stimulated with different concentrations of FBG-C,-R, -W, and -X, or were left unstimulated (−) for 24 h, and TNF (**c**), IL-6 (**d**), and IL-8 (**e**) levels measured by ELISA. Data are shown as mean ± SEM from three independent donors. One-way ANOVA vs. non-stimulated, *p < 0.05, **p < 0.01, ***p < 0.001. **f** 96-well plates were coated with 1 µg ml^−1^ of FBG-C, -R, -W, or -X, or PBS, and incubated with increasing doses of TLR4. Curves were fitted in GraphPad Prism using one-binding site hyperbola equation. Data in the graph are shown as mean ± SEM from four independent experiments

The tenascin family comprises four members: tenascin-C, -R, -W, and -X. Each has a distinct pattern of expression in adults: tenascin-R is predominantly expressed in the central nervous system (CNS), tenascin-W in stem cell niches, and tenascin-X in loose connective tissue. Tenascin-C is unique in that it is not constitutively expressed in most adult tissues but is induced at sites of inflammation^14^. Each tenascin has a similar domain organization (Fig. 1a); all are capped by a C-terminal fibrinogen-like globe (FBG) domain that exhibit a high degree of homology (50–60% sequence identity) (Supplementary Table 1). We previously demonstrated that the FBG domain of tenascin-C (FBG-C) activates TLR4 to drive inflammatory cytokine synthesis in vitro and in vivo^15^, however, it is not known if the FBG domains of the other tenascin family members can induce an immune response.

The FBG domains of tenascin-R, -W, and -X (FBG-R, -W, and -X) were cloned, expressed, and purified as described for FBG-C^15^ (Supplementary Fig. 1). FBG-R and FBG-W induced NF-kB activation in the human monocytic THP1 cell line, as well as IL-6, IL-8, and TNF synthesis in primary human macrophages, to an extent similar to FBG-C. However, FBG-X induced little NF-kB activation and low levels of cytokines (Fig. 1b–e). The activities of FBG-R and -W were TLR4 dependent; being inhibited by a neutralizing TLR4 antibody or by the TLR4 antagonist TAK 242 (Supplementary Fig. 2a–d). To rule out any contribution of contaminating endotoxin in FBG-activated cells, LPS was quantified in FBG preps and levels found to be below the threshold of macrophage detection^15^. In addition, FBG domains were pre-treated with polymyxin B before cell stimulation, which inhibits LPS activity but not that of FBG domains (Supplementary Fig. 2e, f). Moreover, solid phase binding assays demonstrated that FBG-C, -R, and -W bound directly to the TLR4 extracellular domain with similar affinities (*K*_D_ ~50–60 nM), but that FBG-X was unable to bind (Fig. 1f), corroborating the activity observed in cellular assays.

### Three sites in tenascin FBG domains implicated in TLR4 activation

**Fig. 2:**
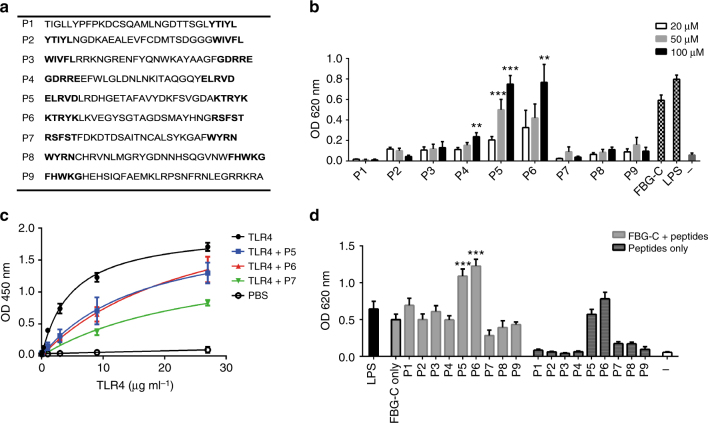
Peptide mapping reveals specific regions in FBG-C involved in TLR4 activation and binding. **a** Nine peptides of ~30 amino acids long from FBG-C were synthesized; overlapping amino acid sequences are shown in bold. **b** THP1 NF-kB cells were stimulated with LPS (0.5 ng ml^−1^), FBG-C (0.5 µM), or 20, 50, or 100 µM of peptides 1–9 for 24 h and NF-kB activation was measured using QUANTI-Blue™. Data shown as mean ± SEM, *n* = 4 independent experiments. One-way ANOVA vs. unstimulated cells. **p < 0.01, ***p < 0.001. **c** Increasing doses of TLR4 were pre-incubated with 200 µM of peptides before adding them to 96-well plates coated with 1 µg ml^−1^ of FBG-C. Curves were fitted in GraphPad Prism using one-binding site hyperbola equation. Data are shown as mean ± SEM, *n* = 3. **d** THP1 NF-kB cells were left unstimulated (−) or pre-incubated with 100 µM peptides prior to stimulation with 0.5 µM of FBG-C for 24 h. NF-kB activation was measured using QUANTI-Blue™. Data shown as mean ± SEM, *n* = 3 independent experiments. Paired *t*-test vs. FBG-C only, *p < 0.05, **p < 0.01, ***p < 0.001

Nine overlapping peptides encompassing the sequence of FBG-C (Fig. 2a and Supplementary Table 2) were used to further map the activity of this domain. Only peptides 5 and 6 induced NF-kB activation in THP1 cells (Fig. 2b) and cytokine synthesis in primary human macrophages, in a TLR4-dependent manner (Supplementary Fig. 3a, b). Scrambled peptides 5 and 6 were not active, and the activity of peptides 5 and 6 was not affected by polymixin B treatment (Supplementary Fig. 3c, d). Pre-incubation of TLR4 with peptides 5, 6, or 7 significantly reduced receptor binding to FBG-C (Fig. 2c, Supplementary Fig. 3e, and Supplementary Table 3). Moreover, peptide 7 reduced FBG-C-mediated NF-kB activation in THP1 cell lines by 57% (*p* = 0.06), while the addition of peptide 5 or 6 with FBG-C had an additive effect on NF-kB activation (Fig. 2d). Together, these data highlight areas within FBG-C that can activate TLR4, as well as areas that contribute to TLR4 binding.

**Fig. 3:**
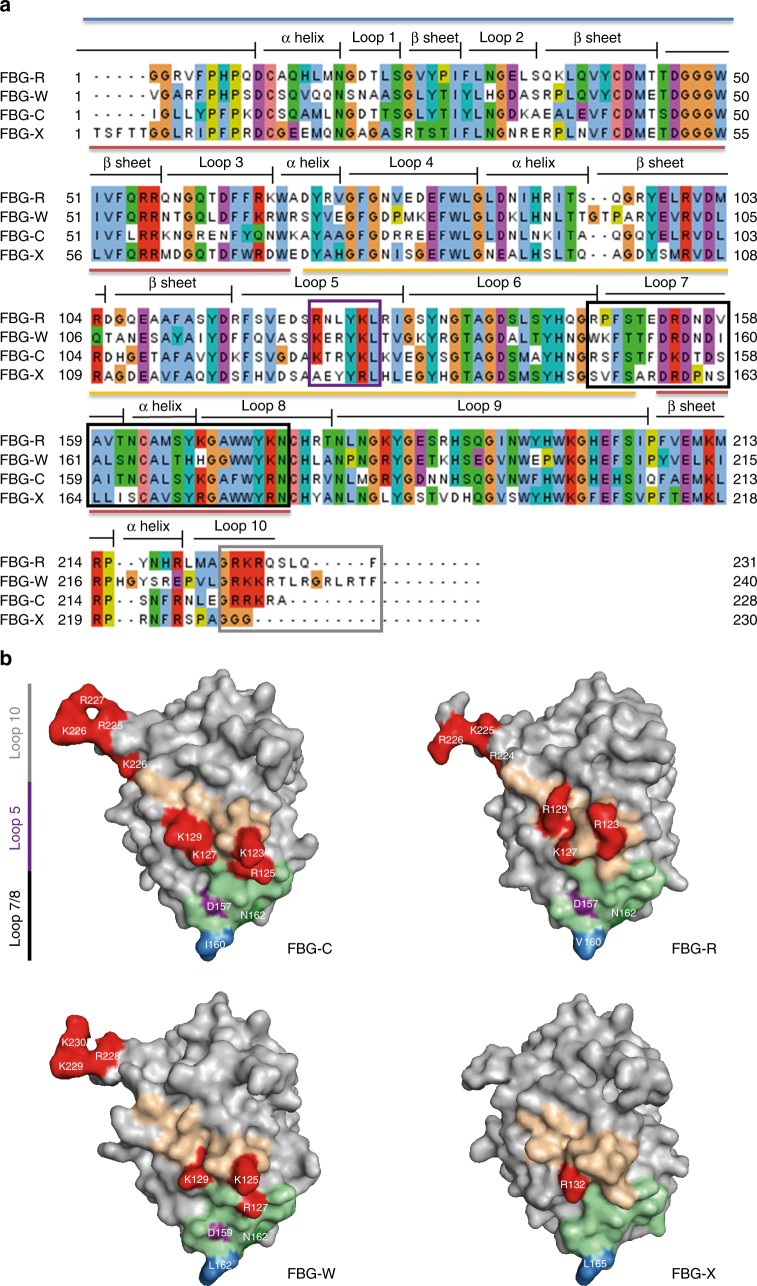
Sequence alignment and homology models of the tenascin family FBG domains. **a** Multiple alignment analysis of the FBG domain of tenascin family members highlighting the secondary structure of the predicted protein models. Blue line indicates the A-subdomain, red line indicates the B-subdomain, and yellow line indicates the P-subdomain. The alignment was colored according to the Clustal color scheme. Light blue: hydrophobic, red: positive charged, green: polar, pink: conserved column of cysteine, violet: negative charged, orange: glycine, yellow: proline, cyan: aromatics. The overlapping sequence of peptides 5 and 6, the sequence of peptide 7, and the C terminus are indicated with purple, black, and gray boxes, respectively. **b** Homology models of the FBG domain of tenascin-C, -R, -W, and -X highlighting the amino acid composition in loop 5, loop 7, and loop 10. The sequence of loop 5 is depicted in pale orange, within which positively charged residues are colored red, any positive residues in the C-terminal loop 10 are also colored red. The sequence of loop 7 is colored pale green within which the triad of polar and hydrophobic residues conserved in FBG-C, -R, and -W but absent in FBG-X, are colored purple, dark green, and blue

Sequence alignment of tenascin FBG domains confirms a high degree of conservation (Fig. 3a), and homology models predict that each exhibits similar subdomain organization and folding (Supplementary Fig. 4a–c). The amino acids comprising peptides 5 and 6 correspond to loop 5 of FBG-C, which protrudes from the surface of subdomain P. The overlapping sequence of peptides 5 and 6 (a series of positively charged residues “KTYRK”), create a cationic ridge containing the sequence KTRYKLK that is present in FBG-C, -R, and -W, but absent from FBG-X (Fig. 3a, purple box, Fig. 3b, and Supplementary Fig. 4a). The amino acids comprising peptide 7 correspond to loop 7, a connecting α-helix and loop 8, also located in the P subdomain. This region is conserved in FBG-C, -R, and -W and in the inactive FBG-X, with the exception of three distinct polar and hydrophobic residues in loop 7 that are absent in FBG-X (Fig. 3a; black box, Fig. 3b, and Supplementary Fig. 4a). Finally, a cluster of positively charged amino acids was observed in the C-terminal loop 10 of FBG-C, -R, and -W that was notably lacking in FBG-X, whose sequence is truncated (Fig. 3a; gray box, Fig. 3b, and Supplementary Fig. 4a).

### Defining the contribution of loops 5, 7, and 10 to TLR4 activation

**Fig. 4:**
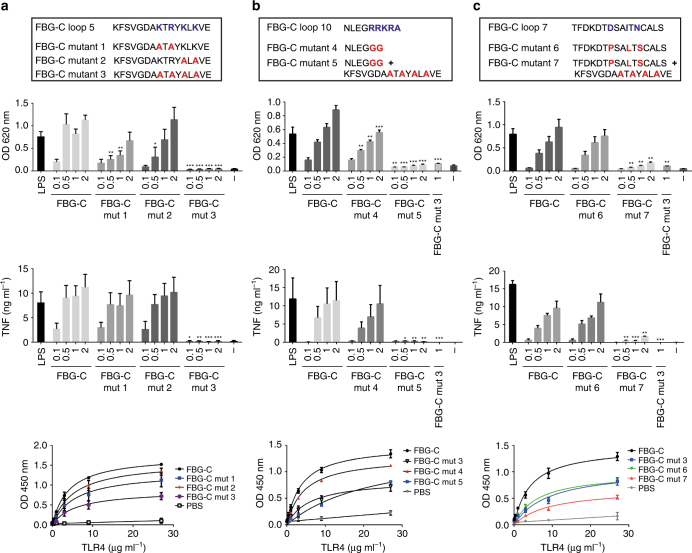
Pinpointing amino acids in loops 5, 7, and 10 of FBG-C that mediate TLR4 binding and activation. Upper panel: Sequences of wild-type FBG-C and mutants 1–7, highlighting wild-type amino acids in blue and mutations in red (loop 5 variants are shown in **a**, loop 10 in **b**, and loop 7 in **c**). Second panel: THP1 NF-kB cells were left unstimulated (−) or stimulated for 24 h with LPS (1 ng ml^−1^), increasing doses (μM) of FBG-C or FBG-C mutants 1–7. NF-kB activation was measured using QUANTI-Blue™. Data shown as mean ± SEM. *n* = 4 independent experiments. Paired *t*-test vs. FBG-C, *p < 0.05, **p < 0.01, ***p < 0.001. Third panel: Primary human macrophages were left unstimulated (−) or stimulated for 24 h with LPS (1 ng ml^−1^), increasing doses (μM) of FBG-C or FBG-C mutants 1–7. Cytokines synthesis was measured by ELISA. Data shown as mean ± SEM. *n* = 4 independent donors. Paired *t*-test vs. FBG-C, *p < 0.05, **p < 0.01, ***p < 0.001. Bottom panel: 96-well plates were coated with 1 µg ml^−1^ of FBG-C or FBG-C mutants 1–7, and TLR4 was added in a dose-dependent manner. Curves were fitted in GraphPad Prism using one-binding site hyperbola equation. Data shown as mean ± SEM; *n* = 4

Peptides were synthesized with alanines substituted for the positively charged residues in loop 5 of FBG-C, and the same mutations were introduced into the FBG-C protein. Peptide and protein variants lacking either the first 2 (mutant 1) or the last two (mutant 2) of the positive residues in the KTRYKLK sequence induced significantly less NF-κB activation and cytokine synthesis compared to wild type, while variants lacking all four charges (mutant 3) exhibited negligible activity (Fig. 4a and Supplementary Fig. 5a, b). The affinity of FBG-C binding to TLR4 was slightly reduced upon loss of two positive charges and a larger, though still modest, reduction was observed upon loss of all four positive charges (Fig. 4a and Supplementary Table 4a). To define the minimum sequence of loop 5 required for TLR4 activation, a panel of peptides was synthesized, ranging from the 5 residue KTYRK sequence alone up to the entire 30 residue loop 5 sequence, in addition to a loop 5 peptide from which KTYRK was deleted (Supplementary Table 2). The two shortest peptides did not induce NF-kB activation, or cytokine synthesis; longer variants were active, however deletion of KTYRK rendered these peptides inactive (Supplementary Fig. 5c). Together, these data indicate that a minimum of three positively charged amino acids in loop 5 are essential for TLR4 activation and that optimal binding to TLR4 requires additional residues outside of loop 5.

To determine whether positive charges in the C-terminal loop 10 of FBG-C contribute to TLR4 activation, two further mutant proteins were synthesized. FBG-C mutant 4 is truncated at residue 223 such that this protein lacks the C-terminal “RRKRA” sequence, while mutant 5 lacks both the C-terminal RRKRA and the four positive charges in loop 5. Mutant 4 exhibited a modest decrease in NF-kB activation and cytokine synthesis, while mutant 5 was not active in these assays, as expected due to the loss of a positively charged loop 5 (Fig. 4b). Mutant 4 exhibited a slight reduction in binding to TLR4, however mutant 5 showed a significant reduction in the affinity for TLR4, which was greater than that observed for FBG-C mutant 3, which lacks only the positively charged loop 5 (Fig. 4b and Supplementary Table 4b). These data suggest that loss of the C-terminal charges alone does not significantly impact the ability of FBG-C to bind to and activate TLR4 but that this region works together with loop 5 to effectively bind to TLR4.

Finally, to determine the contribution of the polar and hydrophobic residues present in loop 7 of FBG-C, FBG-C mutant 6 was created in which three amino acids were substituted for those in FBG-X: D157P, I160L, and N162S. FBG-C mutant 7 contained these three substitutions plus mutation of the four positively charged amino acids in loop 5. Mutant 6 exhibited a modest decrease in NF-kB activation and cytokine synthesis, while mutant 7 was not active in these assays, as expected due to the loss of positive charge in loop 5 (Fig. 4c). FBG-C mutant 6 also exhibited a modest reduction in binding to TLR4, while mutant 7 showed a more significant reduction in the affinity to TLR4, similar to FBG-C mutant 3 (Fig. 4c and Supplementary Table 4c). These data suggest that loop 7 contributes to FBG-C binding to TLR4, and that cooperation between loops 5 and 7 mediates high-affinity binding to this receptor.

### Creating an active FBG-X

**Fig. 5:**
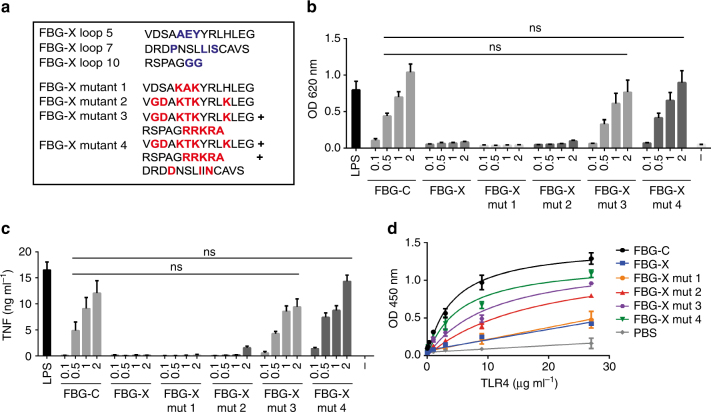
Mutations in FBG-X confer TLR4-activating ability. **a** FBG-X chimeric proteins were designed to introduce the amino acids found in FBG-C to activate and bind to TLR4 (red) into the FBG-X sequence (blue). **b** ThP1 NF-kB cells were left unstimulated (−) or stimulated for 24 h with 0.5 ng ml^−1^ of LPS or increasing doses (μM) of FBG-C, FBG-X, FBG-X mutant 1, 2, 3, and 4. NF-kB activation was measured using QUANTI-Blue™. Data shown as mean ± SEM. *n* = 3 independent experiments. One-way ANOVA vs. FBG-C. **c** Primary human macrophages were left unstimulated (−) or stimulated for 24 h with 1 ng ml^−1^ of LPS or increasing doses (μM) of FBG-C, FBG-X, FBG-X mutant 1, 2, 3, and 4. Cytokine synthesis was measured by ELISA. Data shown as mean + SEM. *n* = 3 independent donors. One-way ANOVA vs. FBG-C. **d** 96-well plates were coated with 1 µg ml^−1^ of FBG-C, FBG-X, FBG-X mutant 1, 2, 3, and 4, and TLR4 was added in a dose-dependent manner. Data shown as mean ± SEM. *n* = 4 independent experiments

To confirm if amino acids identified in FBG-C are sufficient to create an FBG domain capable of binding to and activating TLR4, we generated variants of the inactive FBG-X in which sequences from loops 5, 7, and 10 of FBG-C were substituted into the corresponding region of FBG-X. FBG-X mutant 1 has three amino acids changed to create a positively charged “KAKYR” sequence in loop 5 analogous to the KTRYK sequence in FBG-C, FBG-X mutant 2 has the complete sequence of FBG-C loop 5 inserted, FBG-X mutant 3 includes the mutations made in FBG-X mutant 2 plus addition of the positively charged amino acids at the C terminus, and FBG-X mutant 4 includes mutations in loop 5 and the C terminus, plus the three amino acids substitution in loop 7: P161D, L165I, and S167N (Fig. 5a). Similarly to wild-type FBG-X, FBG-X mutant 1 was unable to activate NF-kB and induce cytokine synthesis, and did not bind to TLR4, while FBG-X mutant 2 exhibited a modest increase in activity at high doses and a low affinity for TLR4 (Fig. 5b, c, d and Supplementary Table 5). These data indicate that while the positive charges in the KTRYK sequence are essential for TLR4 activation they are not sufficient, and that adjacent amino acids in this loop are also required. These data also confirm that additional sites outside loop 5 are required to activate and bind to TLR4 effectively. Both FBG-X mutant 3 and mutant 4 activated NF-kB and induced cytokine synthesis at similar levels to FBG-C. However, mutant 3 bound to TLR4 with a low affinity similar to mutant 2, while mutant 4 bound TLR4 with a high affinity comparable to FBG-C (Fig. 5b–d and Supplementary Table 5). These results confirm that loop 7 and the C terminus of FBG-C can assist loop 5 in binding to TLR4, and highlight specific amino acids that contribute to effective receptor activation. Together, these data demonstrate the cooperative action of three distinct regions in tenascin FBG domains that work together to activate TLR4.

### Identification of related TLR4 agonists

**Fig. 6:**
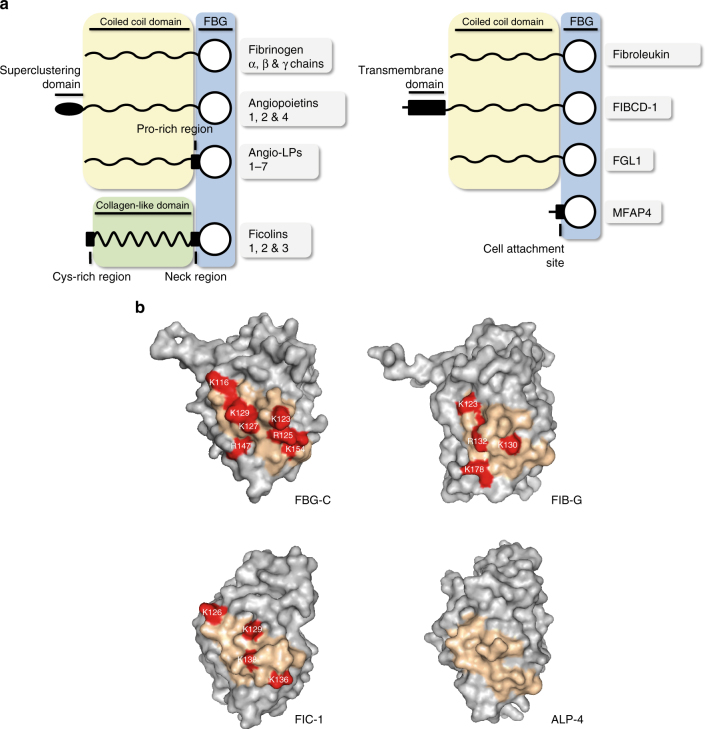
A conserved cationic ridge in fibrinogen-related proteins (FRePs). **a** Simplified domain organization of human FRePs: each protein contains distinct N-terminal sequences but all possess a C-terminal FBG domain, including the four tenascin family members (shown in Fig. 1a), α, β, and γ chains of fibrinogen, the three angiopoietins, seven of the angiopoietin-like proteins (Angio-LPs), the three ficolins, fibroleukin, FIBCD-1, FGL1, and MFAP4. **b** The cationic loop 5 ridge present in FBG-C, -R, and -W, but absent in FBG-X, is conserved in a subset of FRePs, which possess a comparable structural epitope made up of residues from loops 5, 6, and 7. Homology models of the FBG domains of the three FRePs selected for further analysis are shown together with that of tenascin-C (FBG-C); these include two predicted TLR4 agonists; the fibrinogen γ chain (FIB-G) and ficolin-1 (FIC-1), and one FBG domain predicted to be incapable of activating TLR4; angiopoietin-like protein 4 (ALP-4). The region created by residues from loops 5, 6, and 7 on the surface of each FBG domain is shown in pale orange, within which positively charged residues are colored red

Tenascins are not the only proteins that contain an FBG domain. First described in fibrinogen, fibrinogen-related proteins (FRePs) are defined by their possession of this domain. Twenty-four human FRePs exist including fibrinogen chains, tenascins, angiopoietins, angiopoietin-like proteins, ficolins, fibroleukin, fibrinogen-like protein 1 (FGL1), fibrinogen C domain containing 1 (FIBCD-1), and microfibrillar-associated protein 4 (MFAP4) (Fig. 6a)^22^. Each of the human FBG domains exhibits between ~30–40% sequence identity with fibrinogen^23^. However, while the sequences of loops 5, 7, and 10 of FBG-C are extraordinarily well conserved throughout higher eukaryotes (Supplementary Fig. 6a), there is little sequence conservation in these loops among other human FBG domains (Supplementary Fig. 6b). Comparison of the structures of each of these domains, or homology models where crystal structures were not available, demonstrate that each exhibits the same subdomain organization and folding. This analysis also revealed that in some FRePs, a non-contiguous series of positively charged residues derived from loops 5, 6, and 7 come together to create a cationic ridge analogous to that found on the surface of FBG-C, -R, and -W, while other FRePs, like FBG-X, lacked this ridge (Fig. 6b and Table 1). None of the FBG domains possessed an extended cationic C-terminal akin to loop 10 in the active tenascins. A number did contain a triad of polar/hydrophobic residues in loop 7, although in others these residues were absent (Table 1). Based on our data from the tenascin family, we hypothesized that 10 of the other human FBG domains would be capable of activating TLR4 by virtue of possession of a cationic ridge containing three or more positively charged residues.

**Table 1:** Conservation of epitopes involved in TLR4 activation in human FRePs

	Protein	Cationic ridge	Loop 7	Cationic C-terminal	Predicted TLR4 activator
	Tenascin-C	7	+	+	Yes
	Tenascin-R	7	+	+	Yes
	Tenascin-W	6	+	+	Yes
	Tenascin-X	2	−	−	No
*	Angiopoietin-1	4	−	−	Yes
*	Angiopoietin-2	4	−	−	Yes
	Angiopoietin-4	1	−	−	No
	Angiopoietin-like protein 1	5	+	−	Yes
	Angiopoietin-like protein 2	5	−	−	Yes
	Angiopoietin-like protein 3	0	−	−	No
	Angiopoietin-like protein 4	0	−	−	No
	Angiopoietin-like protein 5	2	−	−	No
	Angiopoietin-like protein 6	5	−	−	Yes
	Angiopoietin-like protein 7	2	−	−	No
*	FIBCD-1	2	−	−	No
*	Fibrinogen α chain	2	−	−	No
*	Fibrinogen β chain	3	+	−	Yes
*	Fibrinogen γ chain	4	−	−	Yes
	Fibrinogen-like protein 1	5	−	−	Yes
	Fibroleukin	5	−	−	Yes
*	Ficolin-1	4	−	−	Yes
*	Ficolin-2	2	−	−	No
*	Ficolin-3	2	−	−	No
	MFAP4	2	+	−	No

Comparison of the structures (*crystal available) or homology models of the FBG domains from the 24 human FRePs reveals potential new TLR4 agonists. Cationic ridge: the number of positively charged residues within the region exposed on the surface of the FBG domain around loops 5, 6, and 7. Loop 7: the presence (+) or absence (−) of a triad of polar hydrophobic residues. Cationic C-terminal: the presence (+) or absence (−) of an extended loop 10 containing a cluster of positive charges. FBG domains were designated potential TLR4 activators if they possessed a cationic ridge comprising 3 or more positive charges

**Fig. 7:**
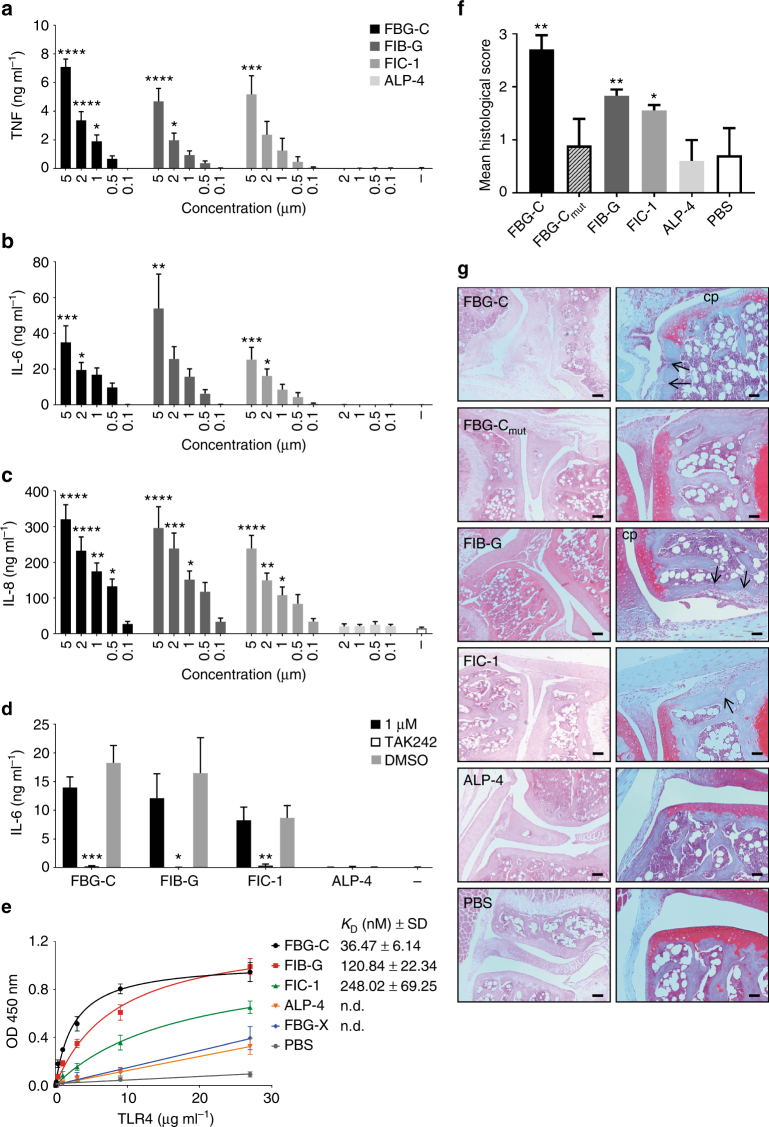
The FBG domains of FIB-G and FIC-1 exhibit pro-inflammatory effects in vitro and in vivo. **a**–**c** Primary human macrophages were stimulated with different concentrations of FBG-C, FIB-G, FIC-1, and ALP-4, or were left unstimulated (−) for 24 h. Cytokine levels were measured by ELISA. Data shown as mean ± SEM from at least three independent donors. One-way ANOVA vs. non-stimulated, *p < 0.05, **p < 0.01, ***p < 0.001, ****p < 0.0001. **d** Primary human macrophages were pre-incubated for 6 h with 3 µM TAK 242 prior to stimulation with FBG-C, FIB-G, FIC-1, and ALP-4 (1 µM), or no stimulation (−) for 24 h. Cytokine synthesis was measured by ELISA. Data shown as mean ± SEM from at least three independent donors. Paired *t*-test vs. non-treated, *p < 0.05, **p < 0.01, ***p < 0.001. **e** 96-well plates were coated with 1 µg ml^−1^ of FBG-C, FBG-X, FIB-G, FIC-1, and ALP-4, or PBS, and incubated with increasing doses of TLR4. Curves were fitted in GraphPad Prism using one-binding site hyperbola equation. Data are shown as mean ± SEM from three independent experiments. **f**, **g** Synovial inflammation was assessed 3 days post injection of each protein (1 µg) or PBS alone into the knees of DBA-1 mice. The histological score was calculated as the mean of seven sections from each knee joint per mouse. *n* = 5 mice per group except for FIC-1 (*n* = 4) (**f**). Mann–Whitney non-parametric test vs. PBS, *p < 0.05, **p < 0.01. Images show representative sections stained by haematoxylin and eosin (left panels) or safranin-O (right panels) (**g**). Mice injected with FBG-C, FIB-G, and FIC-1 exhibit cell infiltration into a thickened synovial lining layer, cellular invasion into the subchondral bone (arrows indicate bone erosion) and loss of articular cartilage proteoglycan (cp), pathological features not observed in mice injected with FBG-C mut or ALP-4.Scale bar left panels: 100 μM, right panels: 50 μM

To test this hypothesis, we selected two FBG domains predicted to activate TLR4; fibrinogen-γ (FIB-G) and ficolin-1 (FIC-1), and one FBG domain predicted to be incapable of activating TLR4; angiopoietin-like protein 4 (ALP-4), and we synthesized each as described for tenascin family domains (Supplementary Fig. 7a–c). Both FIB-G and FIC-1 induced cytokine synthesis in primary human macrophages to an extent similar to FBG-C (Fig. 7a–c), and in a TLR4-dependent manner (Fig. 7d), which was not inhibited by polymyxin B (Supplementary Fig. 7d). However, ALP-4 induced no detectable cytokine synthesis (Fig. 7a–d). Solid phase binding assays confirmed that FIB-G and FIC-1 bound directly to the extracellular domain of TLR4, though with lower affinities than FBG-C, and that ALP-4, like FBG-X, was unable to bind to TLR4 (Fig. 7e). These data support the presence of a cationic ridge in FBG domains as a determinant of TLR4 activation in vitro. We previously demonstrated that intra-articular injection of FBG-C in mice provokes synovial inflammation, along with cartilage and bone destruction^15^. Here we confirm these data, and show that injecting FBG-C mutant 3 (which lacks all four positive charges in the loop 5 KTRYKLK sequence, and which was inactive in vitro), did not elicit inflammation or tissue destruction. FIB-G and FIC-1 also stimulated significant synovial inflammation and tissue destruction, whereas ALP-4 did not (Fig. 7f, g), demonstrating that FBG domains possessing a cationic ridge can stimulate inflammation in vivo, whereas FBG domains lacking a cationic ridge do not.

## Discussion

**Fig. 8:**
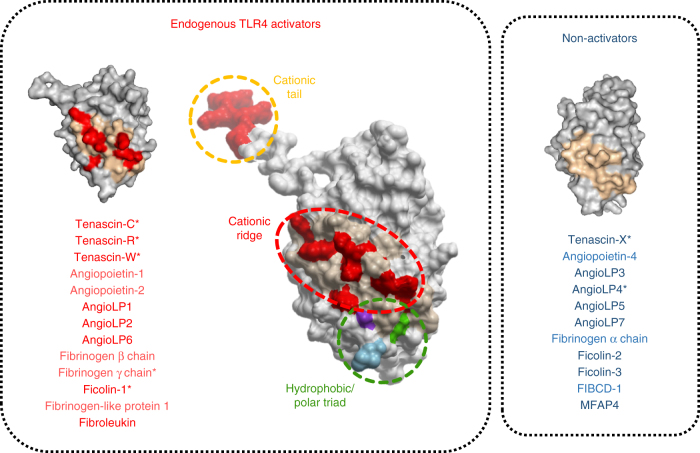
A common danger domain revealed. Three distinct sites within the FBG domain of tenascin-C contribute to TLR4 activation (center panel); a cationic ridge made up of residues from loops 5–7 (pale orange with positive residues highlighted red), underneath which sits a triad of hydrophobic/polar residues from loop 7 (green, purple, and blue), plus a C-terminal cationic tail in loop 10 (positive residues highlighted red). The cationic ridge is the dominant inflammatory epitope; its deletion renders inflammatory stimuli inert and its ectopic expression can convert immunologically inactive proteins into TLR4 agonists. In addition to tenascin-C, in other proteins that contain FBG domains, possession of this inflammatory epitope also confers TLR4-activating capabilities, irrespective of protein family (*denotes validated domains). Together, these data reveal a common mechanism by which distinct inflammatory triggers, spanning a wide range of tissue locations, induced in response to a spectrum of different threats, can activate TLR4 to raise an immune response

Here we show that TLR4 activation is not restricted to the FBG domain of tenascin-C and that conserved domains from other molecules, both within the tenascin family and from diverse protein families, may also act as inflammatory triggers. We demonstrate that TLR4 activation requires a cationic ridge made up of non-contiguous positively charged residues derived from loops 5, 6, and 7 of active FBG domains, and we identify non-essential roles for hydrophobic/polar residues in loop 7, and cationic residues in loop 10, that assist with receptor binding. Finally, our data indicate that this molecular signature creates a TLR4 activation interface that determines whether an FBG domain can be detected by this PRR, or whether an FBG domain will remain immunologically inert (Fig. 8).

In searching for the epitope within tenascin-C that triggers innate immune responses, we identified four novel TLR4 agonists and predict a further 8 likely agonists. Our data are consistent with studies demonstrating that fibrinogen activates TLR4^24–30^, but also provide evidence implicating tenascin-W, tenascin-R, angiopoietins-1 and -2, angiopoietin-like proteins 1, 2, and 6, ficolin-1, FGL1 and fibroleukin in TLR4 activation.

Like tenascin-C, tenascin-W exhibits restricted expression in healthy tissue^31^, and is responsive to inflammatory stimuli^32^. It is also upregulated in many solid tumors, where expression levels are reliable markers of disease detection and prognosis^33–35^. Upregulation of TLR4 in tumors is reported to exhibit both anti- and pro-tumorigenic effects^36^, which are likely dependent on the activating stimuli within the tumor microenvironment. Our data suggest that activation of TLR4 by FBG-W within the tumor stroma could directly affect tumor cell phenotype, and/or the behavior of immune cells resident in the tumor microenvironment, to favor pro-tumoral inflammatory signaling, providing a possible molecular explanation for the association between tenascin-W and poor patient survival. Tenascin-R expression is limited to the CNS, where it is constitutively expressed in the perineuronal nets. Tenascin-R is upregulated at sites of inflammation after neural injury^37–39^ and tenascin-R null mice recover better after spinal cord injury compared to wild-type mice^38^. In vitro, the fibronectin type III repeats (FN6–8) and the EGF-like domain of tenascin-R induce microglial synthesis of chemokine-induced cytokine 3 and TNFα^40^, and the FBG domain enhances microglial adhesion and migration^40^. Our data indicate that FBG-R may also contribute to cytokine synthesis following neural trauma via activation of microglial or astrocytic TLR4. Notably, TLR4 agonism was not confined within specific families of FRePs. For example, the FBG domain of tenascin-X did not activate TLR4. This tenascin is constitutively expressed in connective tissues, such as the dermis and glomeruli, where its role in maintaining tissue architecture, for example, by regulating collagen fibril formation and elastic fiber stability, has been best studied^41^. Tenascin-X expression is regulated upon tissue injury, but unlike other family members, which appear in the early inflammatory stages, tenascin-X appears after the resolution of inflammation during tissue rebuilding^42^. Consistent with these reports, while FBG-X does not activate TLR4, this domain can influence epithelial-to-mesenchymal transition via interaction with the small latent complex of TGF-β, exposing active TGF-β^43^, suggesting that this family member mediates tissue remodeling during repair, rather than inflammation.

Identification of ficolin-1 as a TLR4 agonist was at first glance more surprising; this FReP is best known for triggering complement-mediated inflammation following infection, cell death, or tissue damage^44^. However, compared to its siblings, ficolins-2 and -3, which we predict would not activate TLR4, ficolin-1 circulates at far lower levels in the serum, and is the poorest activator of complement^44^. Instead, it is predominantly expressed by bone marrow-derived myeloid cells, and is found tethered to sialic acid on the surface of monocytes, granulocytes, and activated T cells, where it is incapable of activating complement^45^. These data raise the possibility of an alternative, more localized role for ficolin-1 in activating TLR4 at sites of inflammation. Among our putative TLR4 agonists, angiopoietins-1 and -2, and angiopoietin-like proteins 1 and 2 are known to regulate angiogenesis, while angiopoietin-like protein 6 is linked with lipid metabolism^46, 47^. Fibroleukin was initially identified as a coagulant^48, 49^; and diverse roles for FGL1 have been reported, most recently in the recognition of defective spermatozoa^50^. However, emerging evidence linking angiopoietin-like protein 2 with chronic inflammation in RA, atherosclerosis, diabetes, and cancer^47^, and reports of fibroleukin regulating DC maturation^51^ and macrophage activation^52^, support the idea of immunomodulatory roles for FRePs. While our study offers proof of principle of a molecular tag for TLR4 recognition; FBG domains added exogenously to cells can activate TLR4 in vitro, and cause inflammation in vivo, further work is needed to determine if endogenously expressed FRePs activate TLR4 in situ, and if so, what the physiological and pathological significance of this might be.

The FBG domains of FRePs are defined by a unique structural fold; the N-terminal subdomain A is followed by a central 5 stranded beta sheet making up subdomain B, which is linked to the final subdomain P. However, the third strand of the beta sheet in subdomain B derives from residues that appear sequentially at the end of subdomain P^22^ (Fig. 3a). This unusual globular organization is common to all nine of the human FBG domains crystallized to date (PDB IDs are listed in Supplementary Table 6) and is predicted to be conserved in our homology models of the other 15 FBG domains. The cationic ridge that defines TLR4 recognition is not a linear epitope; it is derived from non-contiguous residues distant in sequence but brought together in the protein by the unique fold of this domain, highlighting the importance of tertiary structure in creating a TLR4 activation epitope. In full-length FRePs, FBG domains are linked to diverse N-terminal sequences; and these can include assembly domains. For example, ficolins form trimers, that further associate into higher-order polymers^53^, tenascins exist as trimers (tenascin-R, -W, and -X) or as hexamers (tenascin-C)^54^, fibrinogen comprises a dimer of three non-identical polypeptide chains (α, β, and γ)_2_^22^, while angiopoietin-like proteins lack an N-terminal super clustering domain that enables the angiopoietins to oligomerize^47^. Our data demonstrate that isolated FBG domains can bind to and activate TLR4, however it does not provide any information about FBG behavior in the context of the whole FReP. While multimerization does not appear to define TLR4 agonism per se, this does not preclude a role for domain oligomerization in TLR4 activation, for example by determining receptor stoichiometry, binding avidity, and internalization or co-receptor recruitment. Biophysical analysis of the mode of TLR4 activation by these agonists should therefore not solely rely on isolated recombinant FBG domains, but include intact fully oligomeric proteins.

It is also important to consider what the physiologically relevant presentation of inflammatory FBG domains might be. Constitutive expression of a number of FRePS that contain TLR4-activating FBG domains raises the related question of how inflammatory activity is regulated. For example, within the complex insoluble structure of the perineural nets, the FBG domain of tenascin-R may be physically constrained in a way that precludes TLR4 binding under normal circumstances. However, these nets are degraded upon injury, which could release FBG-containing fragments capable of TLR4 activation. Alternatively, upregulation of tenascin-R expression upon injury may create tissue concentrations above the threshold of TLR4 detection, or soluble de novo tenascin-R may be more readily accessible to cell surface TLR4. Similar mechanisms may ensure that circulating fibrinogen does not trigger ectopic systemic inflammation; indeed proteinase-dependent fibrinogenolysis is required to activate TLR4-mediated anti-fungal immunity^29^.

Pinpointing sites within FBG-C that are detected by TLR4 highlights a precise target for the amelioration of tenascin-C-driven chronic inflammation; however, the identification of a new subset of protein domains that can activate TLR4 raises more questions than we originally set out to answer. If we now understand more about how endogenous molecules can be marked for innate immune recognition, foremost among the remaining questions must be: why are there so many different proteins with TLR4-activating FBG domains? This may represent physiological economy coupled with intrinsic safeguarding; the fewer endogenous inflammatory epitopes PRRs are alerted by, the smaller the chance of misreading danger signals. The use of a common “danger domain” within a wide range of inflammatory triggers may also serve to alert the immune response to danger at different tissue locations, and in response to different types of threat or cellular stress. Moreover, different FBG domains may induce distinct inflammatory signaling pathways downstream of TLR4 by virtue of different modes of receptor activation or co-receptor use. These data reveal an intriguing new theme underlying the generation of a spectrum of immune responses in the absence of external pathogens.

## Methods

### Reagents

All primers were purchased from Invitrogen. Endpoint Chromogenic LAL Assays and RPMI 1640 were purchased from Lonza, FBS and penicillin/streptomycin were purchased from Gibco. THP1-Blue™ NF-κB cells, Blasticidin, Normocin™, QUANTI-Blue™, human polyclonal (pab-hTLR4) and monoclonal (mabg-hTLR4) TLR4 antibodies, IgG isotype control (pab-sctr) and TAK 242 (CLI-095) TLR4 inhibitor were purchased from InvivoGen. LPS from *E. coli*, Serotype EH100 (Ra) (TLR grade™) was purchased from Enzo life sciences. Polymyxin B was purchased from Fluka. Tetra-his antibody (346700) was purchased from Qiagen. M-CSF was purchased from Peprotech. Rabbit F(ab′)2 anti-mouse IgG-HRP (STAR13B) for use in binding assays, and goat anti-mouse IgG-HRP (170–6516) for use in western blots were purchased from Bio-Rad. Recombinant human TLR4 was purchased from R&D systems. This protein was synthesized using the mouse myeloma NS0-derived cell line, and comprises amino acids Glu24-Lys631, with a C-terminal Ser and 10-His tag. The purity of recombinant TLR4 was confirmed by SDS-PAGE and silver staining (Supplementary Fig. 1d).

### In-silico analysis of homology and protein modeling

All protein sequences were obtained from the UniProt Knowledge database and the accession numbers are presented in Supplementary Tables 7 and 8. Pairwise alignment and multiple protein alignment analysis were performed using Clustal Omega. Alignments were colored using Jalview software. Multiple sequence alignments were performed using Clustal Omega 1.2.1 and colored according to the Clustal color scheme.

Homology models of FBG domains were built using two different methods and two different templates. The homology modeling functionality of the ICM platform was used as follows. The sequence of FBG-C was used to query the PDB for structural homologs using the “PDB Search by Homology” functionality^55^. Matches were triaged by their sequence identity, pP values (a predictive measure of how structurally dissimilar the match is likely to be compared to the query sequence^55^ and the resolution of the structure itself). From this analysis, the structure of fibrinogen-like recognition domain of FIBCD-1 (PDB: 4M7H)^56^ was chosen, presenting a sequence identity of 43% and resolution of 2.0 Å. Sequence alignment of this structure and the tenascin FBG domains was generated using ICM and optimized by hand. Homology modeling was performed within ICM using default parameters. Resultant possible models were scored using an energy-based function and the top 10 superimposed revealing no significant structural difference between them; as such the model with the best score was taken for further analysis. The majority of the sequence was fully built with reference to the template; however, the C-terminal ten residues NFRNLEGRRKR extended beyond the C terminus of the template structures, hence their structure in the model was not be considered to be predictive. Similarly, SWISSMODEL^57^ was used to generate tenascin FBG domain models based on the structure of the C terminus fibrinogen γ chain (PDB: 1FID)^58^. Models generated by either method or template exhibited no significant structural difference. Homology models of each of the other FBG domains were generated using SWISSMODEL. Protein models were colored and structurally analyzed using Pymol Molecular Graphics System, Version 1.7.4 Schrödinger, LLC and ICM-Pro.

### Protein synthesis and biophysical characterization

The FBG domains of tenascin-R, -W, and -X, and of the fibrinogen γ chain, ficolin-1, and angiopoietin-like protein 4, were synthesized and purified as described for the FBG domain of tenascin-C^15^. Briefly, the boundaries of the FBG domain of each protein were determined using alignments published in Uniprot. Each FBG DNA sequence was amplified by PCR using primers specified in Supplementary Tables 9 and 10 and cDNA clones as templates: tenascin-R (ID: 40114168), tenascin-W (ID: 9020609), tenascin-X (ID: 5179997) (IMAGE consortium, Source BioScience); fibrinogen chain γ (ID: 3935073) (MGC Project, GE Healthcare Dharmacon); ficolin-1 (NCBI RefSeq: NM_002003.2) and angiopoietin-like protein 4 (NCBI RefSeq: NM_139314.1) (SinoBiological).

Proteins were expressed in *E. coli* BL21 (DE3) or Rosetta cells and purified using two sequential rounds of Ni2 + chromatography (Bio-Rad). Each column was washed with 100 column volumes of 0.1% Triton-X114 (Sigma-Aldrich, laboratory grade 9036–19–5, lot no. 072k0049) to remove LPS^59, 60^, followed by washing with 100 column volumes of binding buffer, before protein elution using 150 mM imidazole. Proteins were characterized by silver staining and anti-His western blot. Protein secondary structure was analyzed using the J-815 Circular Dichroism Spectrometer (Jasco). The CD spectrum was measured in the “far UV” spectral region (190–250 nm) at 20 °C. Five repeated measurements were taken and the average plotted and analyzed using Spectra Manager™ II software. The LPS content in each protein prep used in this study, including the tenascin family FBG domains (FBG-C, FBG-R, FBG-W, and FBG-X) and the other human FBG domains tested (FIB-G, FIC-1, and ALP-4) was measured using the Endpoint Chromogenic LAL Assays (Lonza) according to the manufacturer’s instructions. Each stock solution of protein was assayed using a protein concentration equivalent to the highest concentration added in cellular assays. Endotoxin values measured typically fall between 2 and 9 pg ml^−1^; if any prep exhibits a value higher than 10 pg ml^−1^ it is discarded and not used in any study.

### Mutagenesis

Changes in the sequences of FBG domains were made using QuikChange II XL Site-Directed Mutagenesis Kit (Agilent Technologies) as described by the manufacturer. Specific primers were designed for each mutation as shown in Supplementary Table 11. Plasmids containing variant PCR products were sequenced (Eurofins, UK) to validate the insertion of the mutations. The clones with the correct mutant sequences were transformed into BL21 DE3 cells for protein over-expression and purification as described above. Proteins were also characterized using SDS-PAGE gels, western blots, CD spectroscopy, and the LPS content was measured.

### Peptides

Peptides synthesized by BioGenes; each was provided at a purity of >90% as a TFA-salt, lyophilized powder. Peptides were reconstituted in sterile PBS and found to be free from LPS using the Endpoint Chromogenic LAL Assays (Lonza) as described above.

### THP1 NF-kB cell culture and stimulation

THP1-Blue™ NF-κB cells (InvivoGen) were cultured in RPMI 1640, 10% FBS, 1% penicillin/streptomycin, 10 µg ml^−1^ blasticidin, and 100 µg ml^−1^ normocin^61^. Cells were plated (10^5^ cells per well) in 96-well plates and stimulated with different doses of LPS, recombinant FBG proteins or peptides. As a control for LPS contamination, the different stimuli were pre-incubated for 30 min with 10 µg ml^−1^ polymyxin B. In the case of co-stimulation with peptides, cells were pre-incubated with 100 µM of different peptides for 30 min at 37 °C and then stimulated with 0.5 µM of FBG-C for 24 h at 37 °C. After 24 h, supernatant was collected and the presence of SEAP was detected using QUANTI-Blue™ (InvivoGen). Absorbance was read at OD 620 nm on FluoStar Omega plate reader.

### Primary human macrophages culture and stimulation

Human monocytes were isolated from peripheral blood (London Blood Bank) by ficoll gradient and counterflow centrifugation. Cells were plated (1 million cells per ml) in RPMI 1640, 5% (v/v) FBS, 1% penicillin/streptomycin and differentiated into macrophages for 5 days with 100 ng ml^−1^ of M-CSF. Macrophages were plated (10^5^ cells per well) in RPMI 1640, 3% (v/v) FBS and 1% penicillin/streptomycin for 24 h before stimulation with different doses of LPS, recombinant FBG proteins or peptides. As a control for LPS contamination, the different stimuli were pre-incubated for 30 min with 10 µg ml^−1^ polymyxin B (final concentration). To assess TLR4 dependency, cells were pre-incubated with 25 µg ml^−1^ PAb-hTLR4 antibody or 25 µg ml^−1^ IgG isotype control for 1 h at 37 °C, or with 3 µM of TAK 242 TLR4 inhibitor for 6 h at 37 °C prior to cell stimulation with 1 ng ml^−1^ of LPS, 1 µM of FBG proteins, or 100 µM of peptides. After 24 h, the medium was collected and cytokines levels detected by ELISA (BD Biosciences) according to the manufacturer’s instructions. Absorbance was read at 450 nm on FluoStar Omega plate reader and data analyzed using Omega software.

### Solid phase binding assay

Nunc, Maxisorp 96-well plates (Thermo Scientific) were coated with 1 µg ml^−1^ solution of recombinant FBG protein in PBS and incubated with shaking overnight at 4 °C. Unspecific binding was blocked with 10% BSA in PBST (0.05% tween20) for 2 h at room temperature. TLR4 was added at a maximum concentration of 27 µg ml^−1^ followed by a series dilution 1:3 in 2% BSA/PBST and incubated for 2 h at room temperature. TLR4 binding was detected using MAb hTLR4 at a final concentration of 1 µg ml^−1^ in 2% BSA/PBST and secondary antibody anti-mouse IgG-HRP (1:1000 dilution) in PBST. Colorimetric reaction was started adding TMB substrate (KPL) and incubating for 10 min in the dark. Color development was stopped using 6% sulfuric acid solution and plates were read at OD 450 nm in FluoStar Omega plate reader. In the case of blocking TLR4-FBG-C binding with peptides, TLR4 was pre-incubated with 200 µM of peptides for 1 h at room temperature.

### Intra-articular injection of FBG domains

*Animals*: Eight-week-old male DBA-1 mice were purchased from Charles River and allowed to acclimatize for 7 days. All animals were fed standard rodent chow and water ad libitum, and were housed (<six mice per cage) in sawdust-lined cages in an air-conditioned environment with 12 h light/dark cycles. All animal procedures were approved by the institutional ethics committee and the UK Home Office.

*FBG injection*: Intra-articular injections were carried out as described previously^15^. Briefly, mice were anesthetized by inhaled isofluorane. Mice were then injected with 1 µg FBG in 10 µl of sterile PBS into the right knee joint using a sterile BD micro-fine 30G insulin syringe. Control mice received an injection of 10 µl PBS alone. At 3 days, mice were killed, and the knee joints were excised and fixed in 10% (v/v) buffered formalin, decalcified, with 10% EDTA and processed to paraffin.

*Histology*: Coronal tissue sections (4 µm) were cut at 7 depths throughout the joint; 80 µm apart and stained with hematoxylin and eosin or Safranin-O to assess joint pathology. Each joint was scored as described^62^: 0 = normal; 1 = cell infiltration with no signs of joint erosion; 2 = inflammation with the presence of erosions limited to discrete foci; and 3 = severe and extensive joint erosion with loss of architecture. Histological analysis was performed by investigators blinded to the experimental groups. The mean score for each animal in an experimental group was calculated by averaging the histopathologic scores in at least five section depths per joint.

### Statistics

The data mean ± SEM or SD and statistical analysis were calculated using GraphPad Prism 6 software. Multiple group means were analyzed by one-way analysis of variance, followed by the Dunnett multiple comparisons test, where appropriate. Two-tailed, paired *t*-test was used for experiments involving only two groups. In vivo data were analyzed by a Mann–Whitney non-parametric test. *P* values lower than 0.05 were considered significant.

### Data availability

The data that support the findings of this study are available from the corresponding author on request.

## Electronic supplementary material

Supplementary information
