# Supplementary material for: Mapping tenascin-C interaction with toll-like receptor 4 reveals a new subset of endogenous inflammatory triggers
Source: Nat Commun. 2017 Nov 17;8:1595. doi: 10.1038/s41467-017-01718-7 (PMC5693923; doi:10.1038/s41467-017-01718-7)
Supplement: Supplementary file 1 — Supplementary information [file 41467_2017_1718_MOESM1_ESM.pdf]

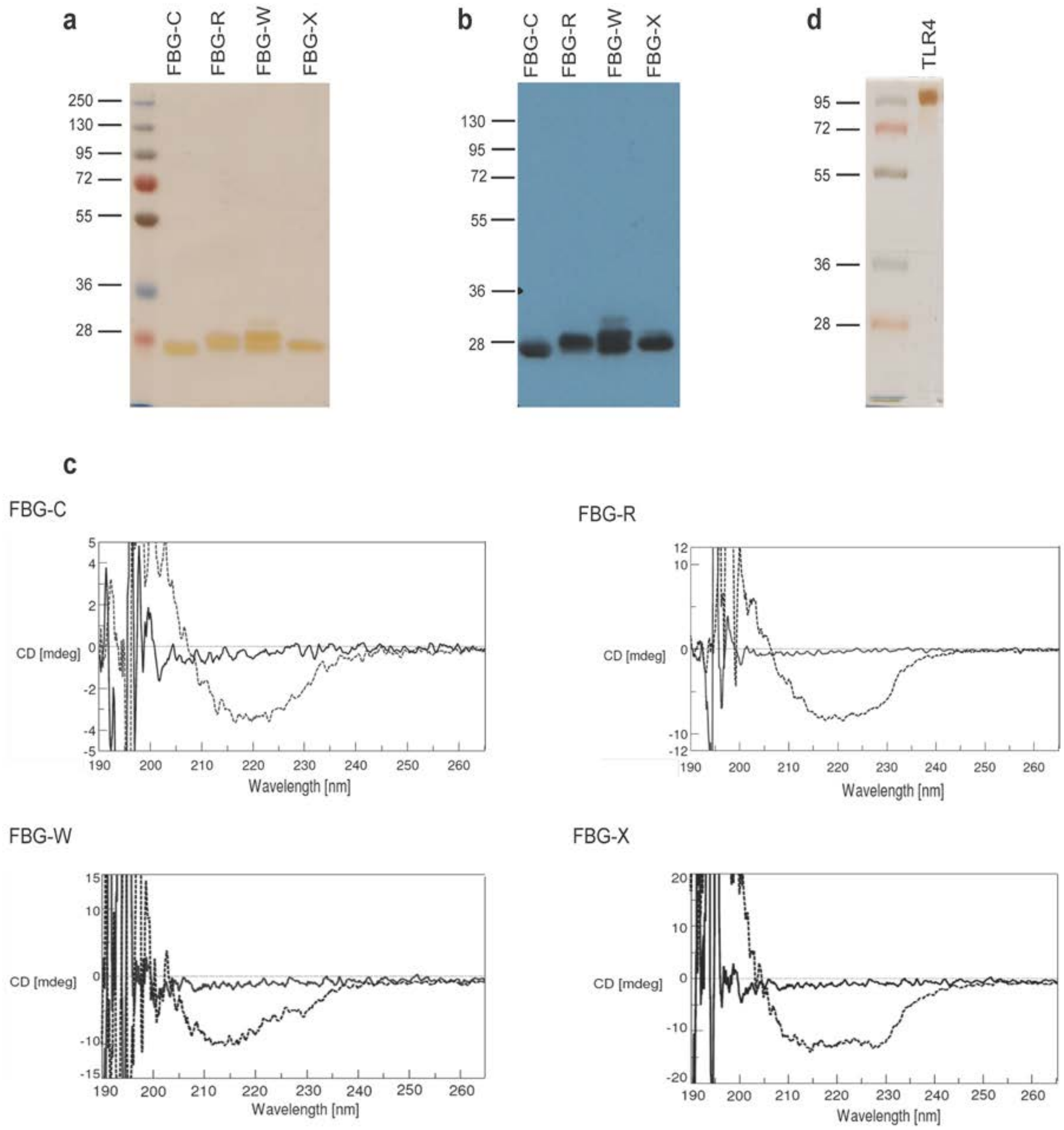

Supplementary Figure 1. Biophysical characterization of FBG-C, -R, -W –X. a. Protein purity was verified by silver staining of 1 μg of FBG-C, -R, -W and –X. **b.** Anti-His tag western blot of 1 μg of FBG-C, -R, -W and –X was used to confirm protein identity. **c.** Circular dichroism (CD) spectra in the far UV region of FBG-C, -R, -W and –X was used to show similar folding profiles for each. The buffer control signal is shown as a black solid line and the FBG-C, -R, -W and –X negative peaks as black dashes. **d.** Protein purity was verified by silver staining of 1 μg of recombinant human TLR4 from R&D systems.

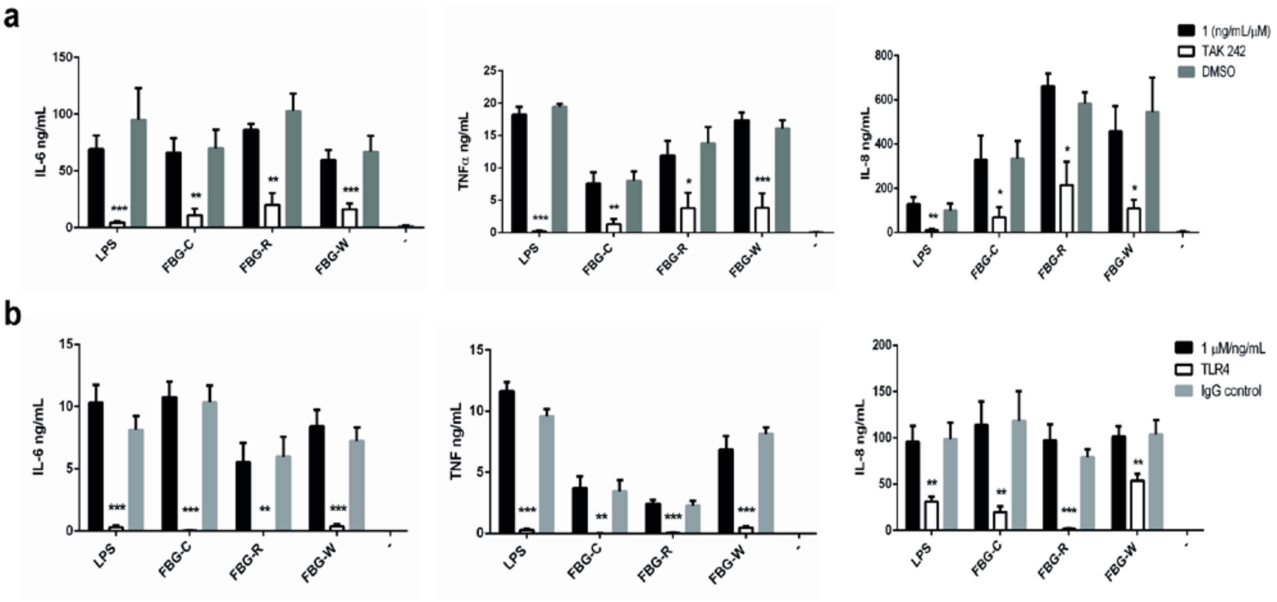

c

Stimulus	IL-6	IL-8	TNF
LPS	93.59 ± 1.48	91.97 ± 3.68	98.97 ± 0.80
FBG-C	83.53 ± 12.14	79.22 ± 9.88	82.95 ± 17.84
FBG-R	76.95 ± 19.13	67.72 ± 19.36	68.23 ± 17.41
FBG-W	72.85 ± 23.93	76.18 ± 9.25	78.00 ± 16.42

d

Stimulus	IL-6	IL-8	TNF
LPS	97.31 ± 2.08	67.58 ± 5.64	97.83 ± 1.05
FBG-C	99.84 ± 0.09	82.71 ± 6.59	99.78 ± 0.43
FBG-R	100 ± 0.00	98.82 ± 1.80	98.48 ± 1.30
FBG-W	95.54 ± 4.17	47.39 ± 12.36	93.15 ± 2.65

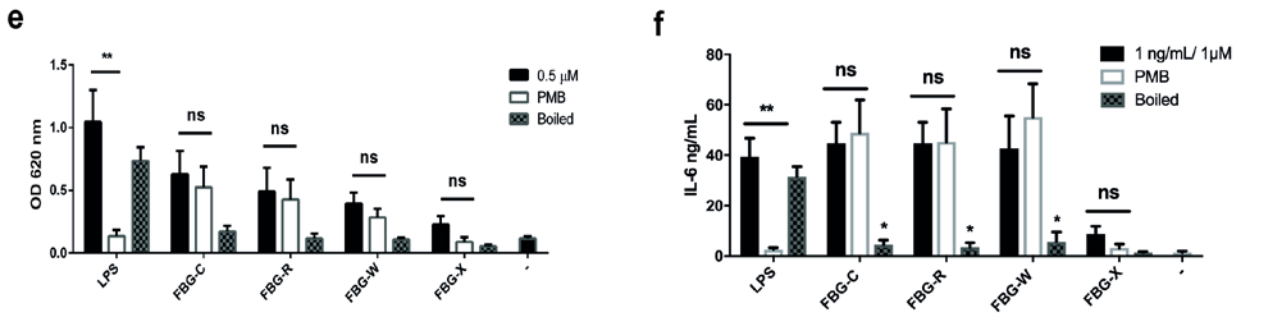

Supplementary Figure 2. Cytokine induction by FBG-C, -R and –W is TLR4 dependent and not due to LPS contamination. **a, b.** Primary human macrophages were pre-incubated for 6 h with 3μM TLR4 inhibitor TAK242 (**a**) or for 30 min with 25 μg mL^{-1} of TLR4 polyclonal antibody (**b**) prior to stimulation with LPS (1 ng mL^{-1}) or FBG-C, -R and –W (1 μM), or no stimulation (-) for 24h. Data are shown as mean ± SEM from 3 independent donors. Paired t-test vs non-treated with TAK242 or TLR4 antibody, *p<0.05, **p<0.01, ***p<0.001. **c.** % of inhibition ± SEM of LPS, FBG-C, -R and -W activity by TLR4 inhibitor TAK242. **d.** % of inhibition ± SEM of LPS, FBG-C, -R and -W activity by TLR4 antibody. **e, f.** THP1 NF-κB cells (**e**) or primary human macrophages (**f**) were left unstimulated (-) or stimulated with LPS (1ng ml^{-1}) or FBG-C, -R, -W and –X (1 μM) incubated for 30 min with polymyxin B (PMB) or boiled for 15 min. NF-κB activation was measured after 24 h using QUANTI-Blue[TM]. Cytokine synthesis was measured by ELISA Data shown as mean ± SEM, N=3 independent experiments. Paired t-test PMB or boiled vs non- treated, *p<0.05

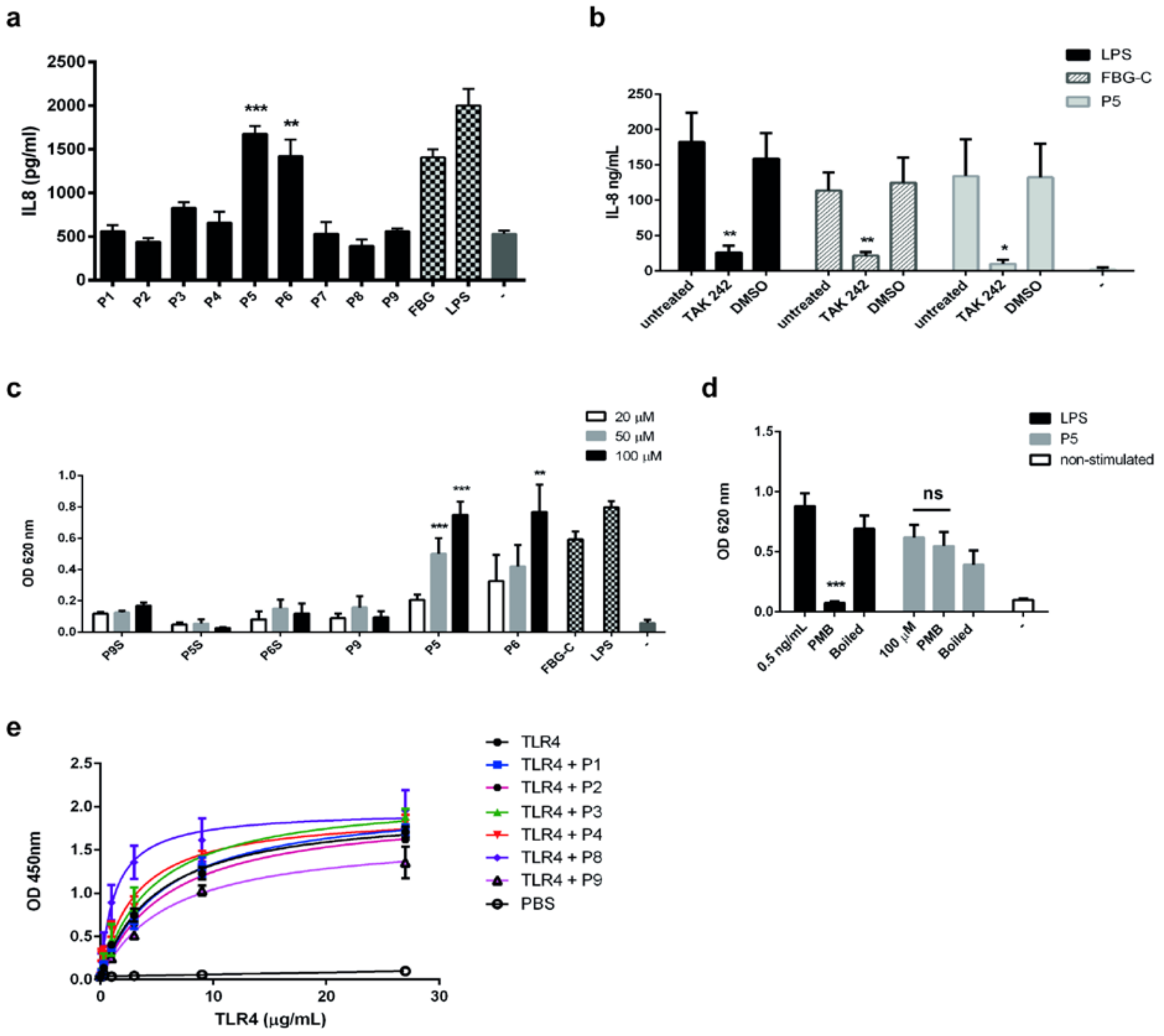

Supplementary Figure 3. Peptides 5 and 6 can induce NF-kB and cytokine synthesis in a TLR4 dependent manner. a. Primary human macrophages were left unstimulated (-) or stimulated with LPS (1 ng mL^{-1}), FBG-C (1 µM) or 20 µM of peptides 1-9 for 24h and cytokine synthesis was measured by ELISA. **b.** Primary human macrophages were pre-incubated for 6 h with 3 µM TAK242 or DMSO, prior to stimulation with 1ng mL^{-1} of LPS, 1 µM of FBG-C or 100 µM of peptide 5. Cytokine synthesis was measured after 24h by ELISA. **c.** THP1 NF-kB cells were left unstimulated (-) or stimulated with 20, 50 or 100 µM of peptides or scrambled peptide controls for 24 h and NF-kB activation was measured using QUANTI-Blue™. **d.** THP1 NF-kB cells were left unstimulated (-) or stimulated with 0.5 ng mL^{-1} of LPS or 100 µM of peptide 5 for 24 h. Samples were previously incubated for 30 min with 10 µg ml^{-1} polymyxin B or boiled for 15 min. NF-kB activation was measured using QUANTI-Blue™. Data are shown as mean ± SEM, N=3 independent experiments or 3 independent donors. One-way ANOVA vs unstimulated cells or paired t-test vs non-treated, *p<0.05, **p<0.01, ***p<0.001.**e.** Increasing doses of TLR4 were pre-incubated with 200 µM of peptides before adding them to 96 well plates coated with FBG-C. Curves were fitted in GraphPad Prism using one binding site hyperbola equation. Data are shown as mean ± SEM, N=3.

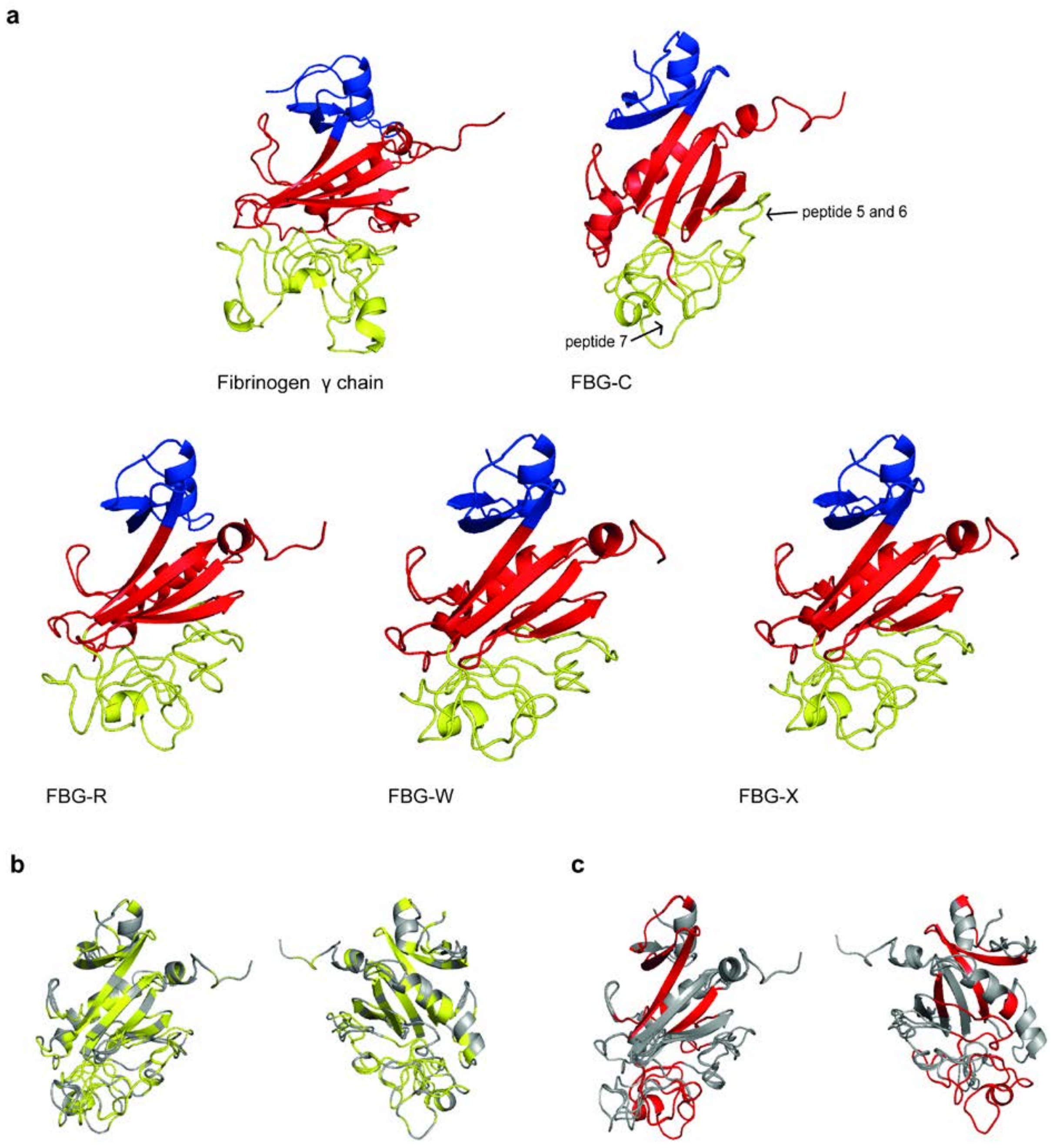

Supplementary Figure 4. Modelling the structure of FBG-C, -R, -W and –X. a. The structures of FBG-C, -R, -W and –X were modelled based on the C-terminal fibrinogen γ chain or the FIBDC crystal structure (FIBDC not shown). Sub-domain protein organisation is highlighted: A-subdomain in blue, B-subdomain in red and P-subdomain in yellow. The location of peptides 5, 6 and 7 are indicated by the arrows in the structure of the FBG-C model. **b.** FBG-C, -R, -W and –X models were aligned and coloured using PyMOL Molecular Graphics System highlighting in yellow conserved amino acid sequences. Figure on right panel rotated 180° compared to figure on the left. **c.** FBG-C, -R, -W and –X models were aligned and coloured using PyMOL Molecular Graphics System highlighting in red conserved structure. Figure on right panel rotated 180° compared to figure on the left.

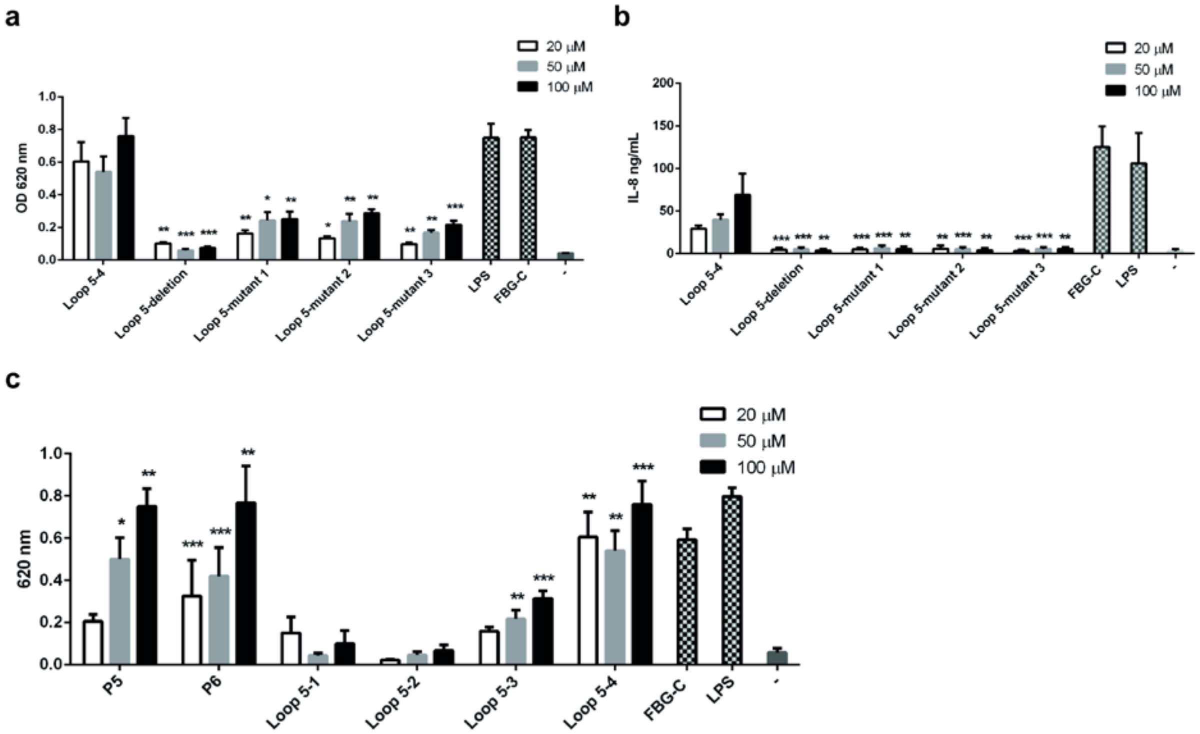

Supplementary Figure 5. Peptide variants of loop 5 can induce NF-kB activation. a. THP1 NF-kB cells were unstimulated (-) or stimulated with 20, 50 or 100 µM of different peptide versions of loop 5 for 24 h and NF-kB activation was measured using QUANTI-Blue™ . Data shown as mean ± SEM, N=3 independent experiments. One-way ANOVA vs unstimulated cells. **b.** Primary human macrophages were left unstimulated or stimulated for 24h with 20, 50 and 100 µM of loop 5-4, loop 5-mutation 1, loop 5-mutation 2 and loop 5 mutation-3. IL-8 synthesis was measured by ELISA. Data shown as mean ± SEM. N=3 independent donors. Paired t-test vs loop 5-4, *p<0.05, **p<0.01, ***p<0.001. . **c**. THP1 NF-kB cells were left unstimulated (-) or stimulated with 20, 50 and 100 µM of peptides loop 5-4, loop 5-deletion, loop 5-mutation 1, loop 5-mutation 2 and loop 5 mutation-3. NF-kB activation was measured after 24h using QUANTI-Blue™. Data shown as mean ± SEM. N=3 independent experiments. Paired t-test vs loop 5-4, *p<0.05, **p<0.01, ***p<0.001.

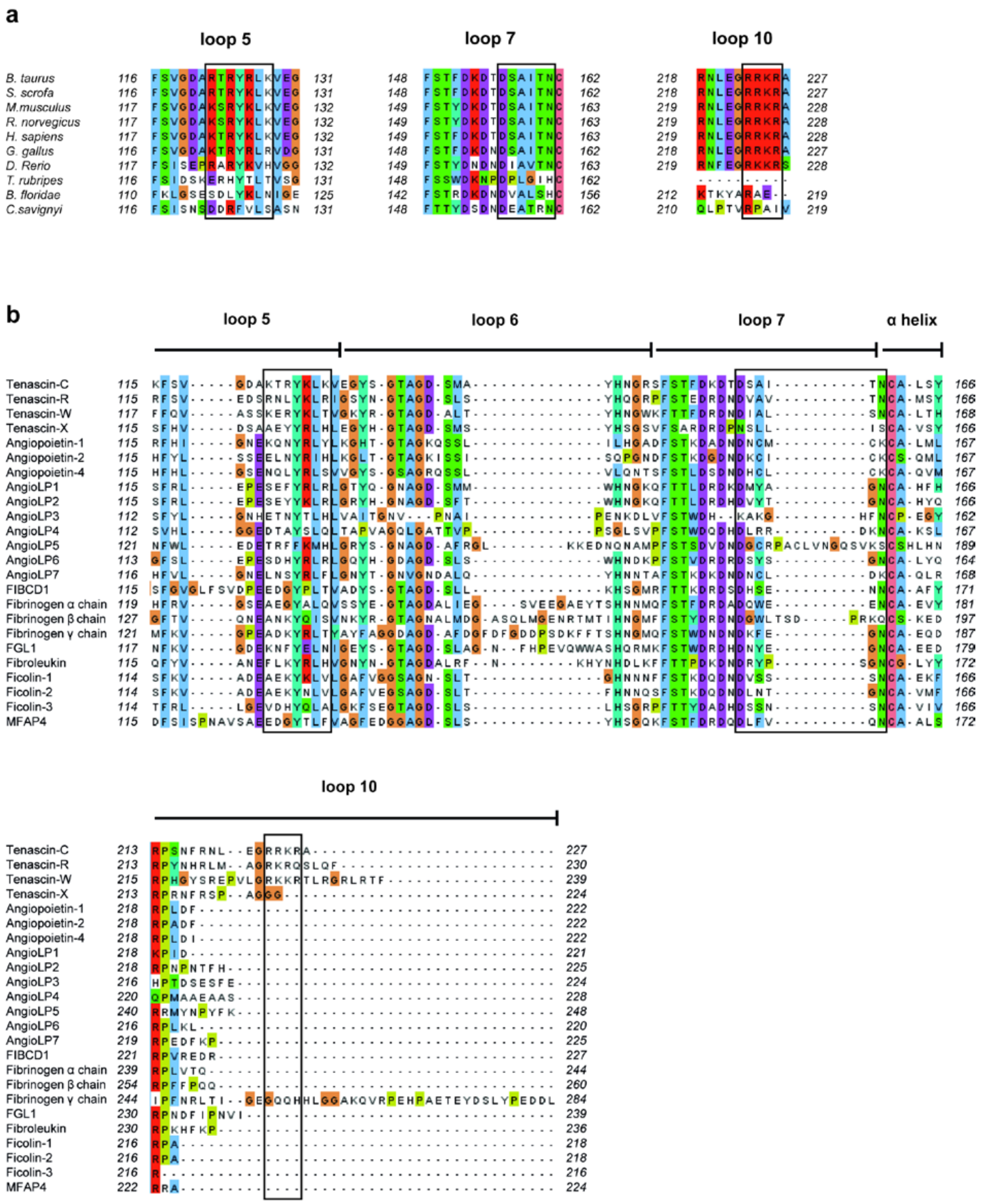

Supplementary Figure 6. The linear sequences of loops 5, 7 and 10 of FBG-C are not conserved in human FRePs. a. Multiple sequence alignment of the FBG domain of tenascin-C of different species. **b.** Multiple sequence alignment of human FBG domains. The amino acids involved in TLR4 activation and binding in loop 5, 7 and 10 are highlighted in a black box. The alignment was coloured according to the Clustal colour scheme. Light blue: hydrophobic. Red: positive charged. Green: Polar. Pink: conserved column of cysteine. Violet: negative charge. Orange: glycine. Yellow: proline. Cyan: aromatics.

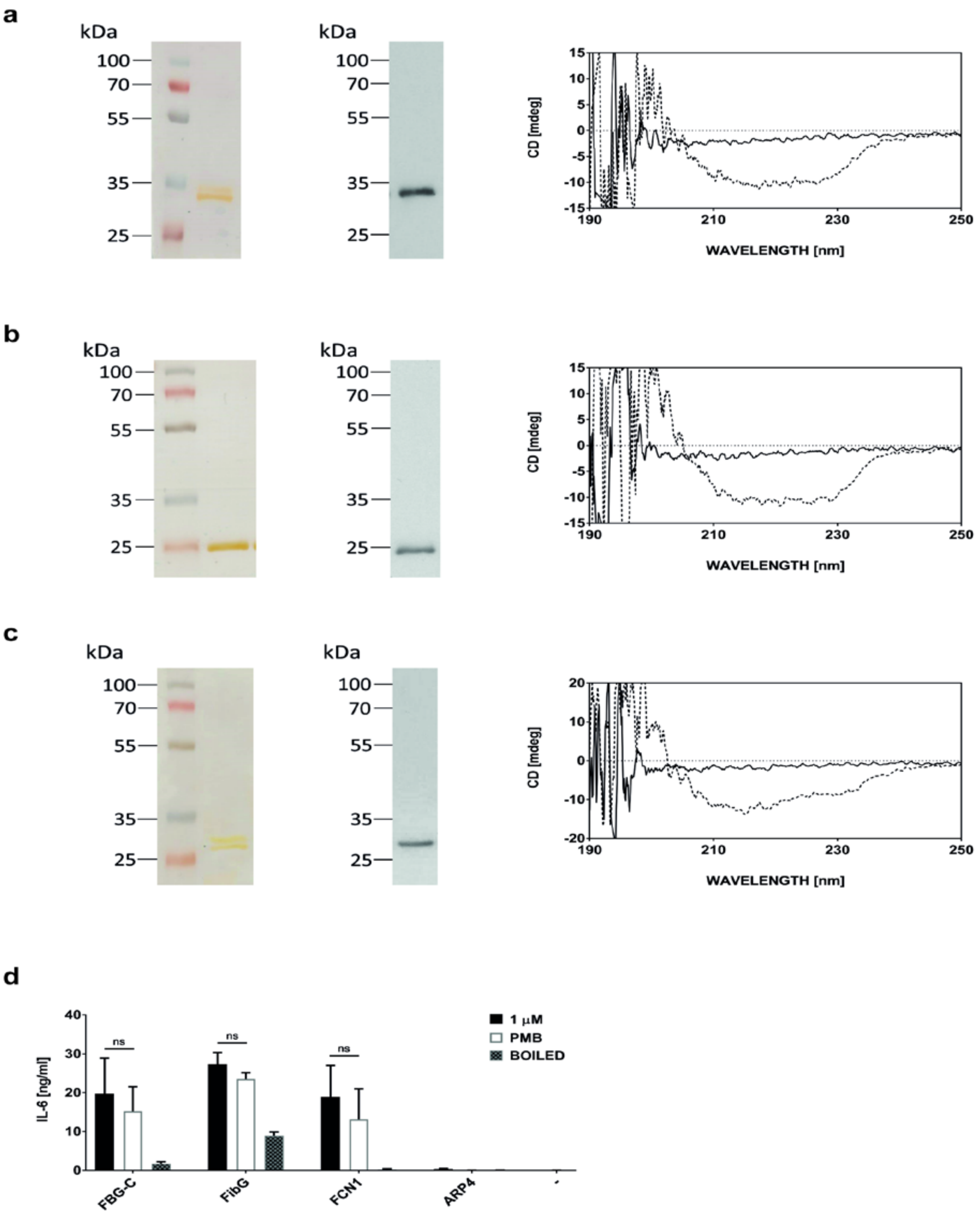

Supplementary Figure 7. Synthesis of and cytokine induction by FIB-G, FIC-1 and ALP-4. Protein purity was verified by silver staining of 1 µg of FIB-G (**a**), FIC-1 (**b**) and ALP-4 (**c**)(left panel). Anti-His tag western blot of 1 µg of each protein was used to confirm protein identity (middle panel)**.** Circular dichroism (CD) spectra in the far UV region were used to show similar folding profiles for each. The buffer control signal is shown as a black solid line and the FIB-G, FIC-1 and ALP-4 negative peaks as black dashes (right panel). **d.** Primary human macrophages were stimulated with FBG-C, FIB-G, FIC-1 and ARP-4 (1 µM) incubated for 30 min with polymyxin B (PMB) or boiled for 30 min. IL-6 synthesis was measured by ELISA. Data shown as mean ± SEM from 3 independent donors. Paired t-test vs non-treated, ns, not significant.

Supplementary Table 1. Percentage of identity amongst the amino acid sequences of the FBG domains of human tenascin-C, -R, -W and -X (FBG-C, -R, -W, –X)

	FBG -C	FBG -X	FBG -R
**FBG-X**	52.89	-	-
**FBG-R**	59.65	54.67	-
**FBG-W**	53.02	49.56	55.74

Supplementary Table 2. Amino acid sequences of the peptides used in this study.

**P1**	TIGLLYPFPKDCSQAMLNGDTTSGLYTIYL
**P2**	YTIYLNGDKAEALEVFCDMTSDGGGWIVFL
**P3**	WIVFLRRKNGRENFYQNWKAYAAGFGDRRE
**P4**	GDRREEFWLGLDNLNKITAQGQYELRVD
**P5**	ELRVDLRDHGETAFAVYDKFSVGDAKTRYK
**P6**	KTRYKLKVEGYSGTAGDSMAYHNGRSFST
**P7**	RSFSTFDKDTDSAITNCALSYKGAFWYRN
**P8**	WYRNCHRVNLMGRYGDNNHSQGVNWFHWKG
**P9**	FHWKGHEHSIQFAEMKLRPSNFRNLEGRRKRA
**P5S**	KGVFLTRYVTDARDVHFDKYGASRELEAKD
**P6S**	YKGTSDHFRVGSNSRYETSMGKGAATLKY
**P9S**	AFERHMKWKRKLRGAGHREPELHSNSNRIFQF
**Loop 5-1**	**KTRYK**
**Loop 5-2**	GDA**KTRYKLK**VEG
**Loop 5-3**	DKFSVGDA**KTRYKLK**VEGYSG
**Loop 5-4**	AFAVYDKFSVGDA**KTRYK**LKVEGYSGTAGD
**Loop 5-deletion**	GETAFAVYDKFSVGDAVEGYSGTAGDSMAY
**Loop 5-mutant 1**	AFAVYDKFSVGDA**A**T**A**YKLKVEGYSGTAGD
**Loop 5-mutant 2**	AFAVYDKFSVGDAKTRY**A**L**A**VEGYSGTAGD
**Loop 5-mutant 3**	AFAVYDKFSVGDA**A**T**A**Y**A**L**A**VEGYSGTAGD

Nine peptides (P1-9) were used to map the FBG-C active site; together they span the whole sequence of FBG-C with each peptide containing a sequence that overlaps with the contiguous peptide. Scrambled versions of peptides 5, 6 and 9 (P5S, P6S, P9S) were made as controls. Peptides comprising loop 5 of FBG-C and adjacent amino acids were made including deletion and mutations in this amino acid sequence.

Supplementary Table 3. Peptides block TLR4-FBG-C binding.

Protein	KD (nM) ± SD	p value
TLR4 only	68.85 ± 7.79	
TLR4 + P1	78.75 ± 11.33	0.280
TLR4 + P2	83.29 ± 5.24	0.056
TLR4 + P3	64.45 ± 15.67	0.672
TLR4 + P4	42.63 ± 10.06	0.023 *
TLR4 + P5	241.22 ± 89.15	0.028 *
TLR4 + P6	370.82 ± 141.64	0.021 *
TLR4 + P7	383.71 ± 113.31	0.008 **
TLR4 + P8	31.87 ± 8.22	0.048 *
TLR4 + P9	86.26 ± 16.57	0.17

Increasing doses of TLR4 were pre-incubated with 200 µM of peptides before adding them to 96 well plates coated with FBG-C. The affinity of FBG-C binding to TLR4 was calculated using GraphPad Prism using one binding site hyperbola equation, N=3. Un-paired t-test vs TLR4 only. *p<0.05, ** p<0.01.

Supplementary Table 4. Affinity of TLR4 binding to FBG-C mutants.

a.

Protein	KD (nM) ± SD	p value
FBG-C	58.21 ± 5.24	
FBG-C mut 1	81.01 ± 15.45	0.031 *
FBG-C mut 2	75.92 ± 15.58	0.074
FBG-C mut 3	121.10 ± 15.29	0.0002 ***

b.

Protein	KD (nM) ± SD	p value
FBG-C	53.4 ± 7.93	
FBG-C mut 4	63.03 ± 8.92	0.234
FBG-C mut 5	336.4 ± 103.12	0.009 **

c.

Protein	KD (nM) ± SD	p value
FBG-C	60.48 ± 11.43	
FBG-C mut 6	86.69 ± 21.25	0.135
FBG-C mut 7	123.78 + 28.33	0.023 **

96 well plates were coated with FBG-C or with FBG-C mutants, and TLR4 was added in a dose dependent manner. The affinity of each FBG variant for TLR4 was calculated using GraphPad Prism using one binding site hyperbola equation. N=4 for FBG-C, FBG-C mutant 1, 2 and 3 (a). N=3 for FBG-C, FBG-C mutant 4, 5, (b) 6 and 7 (c). Un-paired t-test vs FBG-C, *p<0.05, **p<0.01, ***p<0.001.

Supplementary Table 5. Affinity of TLR4 binding to FBG-X mutants.

**Protein**	**KD (nM) ± SD**	**p value**
FBG-C	59.77 ± 7.08	
FBG-X	ND	
FBG-X mutant 1	ND	
FBG-X mutant 2	173.09 ± 56.66	0.007**
FBG-X mutant 3	154.96 + 46.74	0.006**
FBG-X mutant 4	78.33 + 18.41	0.109

96 well plates were coated with FBG-C, FBG-X, FBG-X mutants 1, 2, 3 or 4, and TLR4 was added in a dose dependent manner. The affinity of FBG-C or FBG-X mutants to TLR4 was calculated using GraphPad Prism using one binding site hyperbola equation. N=4. Un-paired t-test vs FBG-C. **p<0.01.

Supplementary Table 6. FBG domain crystal structures

Protein	PDB ID
Angiopoietin 1	4EPU
Angiopoietin 2	1Z3U
FIBCD-1	4M7H
Fibrinogen α chain	1FZD
Fibrinogen β chain	1FZA
Fibrinogen γ chain	1FID
Ficolin-1	2JHM
Ficolin-2	4R9J
Ficolin-3	2J5Z

Supplementary Table 7. The name and accession numbers of the 24 human FRePs

Protein	Accession no.
Angiopoietin 1	Q15389
Angiopoietin 2	O15123
Angiopoietin 4	Q9Y264
Angiopoietin-like protein 1	O95841
Angiopoietin-like protein 2	Q9UKU9
Angiopoietin-like protein 3	Q9Y5C1
Angiopoietin-like protein 4	Q9BY76
Angiopoietin-like protein 5	Q86XS5
Angiopoietin-like protein 6	Q8NI99
Angiopoietin-like protein 7	O43827
Fibrinogen α chain	P02671
Fibrinogen β chain	P02675
Fibrinogen γ chain	P02679
Fibrinogen-like protein 1	Q08830
Fibroleukin	Q14314
FIBCD-1	Q8N539
Ficolin-1 (M)	O00602
Ficolin-2 (L)	Q15485
Ficolin-3 (H)	O75636
MFAP4	P55083
Tenascin-C	P24821
Tenascin-R	Q92752
Tenascin-W	Q9UQP3
Tenascin-X	P22105

Supplementary Table 8. The accession numbers of tenascin-C from different species

Protein	Accession number
S. scrofa	Q29116
M. musculus	Q80YX1
R. norvegicus	B2LYI9
B. taurus	A0JN60
G. gallus	P10039
D. rerio	Q4FAI8
T. rubripes	Q7SZG1

Supplementary Table 9. Forward and reverse primer sequences for tenascin-R, -X and –W FBG domains.

Gene	Accession number	Basepair number of FBG	Domain boundaries	Primer	Sequence	Size of PCR product
TNR	NM_003285.2	696	3937-4632	TNR FBG Forward	GCC **CAT ATG** CAT CAT CAT CAT CAT CAT GGA GGC CGG GTG TTC CCT CAT	735
				TNR FBG Reverse	GCC **CTC GAG** TTA GAA CTG TAA GGA CTG CCG TTT TCT	
TNX	NM_019105.6	693	12239-12931	TNX FBG Forward	GCC **CAT ATG** CAT CAT CAT CAT CAT CAT ACCTCTTTCACCACGGGT	732
				TNX FBG Reverse	GCC **CTC GAG** TTA GCC TCC CCC CGC TGG GGA G	
TNW	NM_022093.1	723	3291-4013	TNW FBG Forward	GCC **CAT ATG** CAT CAT CAT CAT CAT CAT GTT GGT GCC CGT TTC CCA	762
				TNW FBG Reverse	GCC **CTC GAG** TTA GAA CGT TCG CAG CCT TCC TCT CAG	

Each sequence has a His-tag (underlined) and a restriction site in the forward primer for *Nde*I (bold) and in the reverse primer for *Xho*I (bold).

Supplementary Table 10. Forward and reverse primer sequences FBG domains from the fibrinogen chain γ, ficolin-1 and angiopoietin-like protein 4.

Gene	Accession number	Basepair number of FBG	Domain boundaries	Primer	Sequence	Size of PCR product
FGG	BC021674.1	807	556-1362	FGG FBG Forward	CAC **CCA TGG** CAC ATC ATC ATC ATC ATC ATC AAA TCC ATG ATA TCA CTG G	845
				FGG FBG Reverse	GTT **CTC GAG** TTA AAC GTC TCC AGC CTG TTT GGC TCC C	
FCN1	NM_002003.2	658	362-1019	FCN1 FBG Forward	CAC **CAT ATG** CAT CAT CAT CAT CAT CAT CAG TCG TGT GCG ACA GGC CCA C	694
				FCN1 FBG Reverse	GCT **CTC GAG** TTA GGC GGG CCG CAC CTT CAT CTC TG	
ANGPTL4	NM_139314.1	687	730-1416	ANGPTL4 FBG Forward	AAA **CAT ATG** CAT CAT CAT CAT CAT CAT AGC CGC CTG CAC CGG CTG C	723
				ANGPTL4 FBG Reverse	ACT **CTC GAG** TTA GGA GGC TGC CTC TGC TGC CAT G	

Each sequence has a His-tag (underlined) and a restriction site in the forward primer for *NcoI* (fibrinogen chain γ, bold) or *NdeI* (ficolin-1 and angiopoietin-like protein 4, bold), and in the reverse primer for *XhoI* (bold).

Supplementary Table 11. Primer sequences used to insert specific mutations in FBG-C and FBG-X.

Protein	Primer sequence	
**FBG-C mutant 1**	Primer Forward	GTG GGA GAT GCC GCG ACT GCC TAC AAG CTG AAG GTG GAG
	Primer Reverse	CAC CTT CAG CTT GTA GGC AGT CGC GGC ATC TCC CAC GC
**FBG-C mutant 2**	Primer Forward	GCC AAG ACT CGC TAC GCG CTG GCG GTG GAG GGG TAC
	Primer Reverse	GTA CCC CTC CAC CGC CAG CGC GTA GCG AGT CTT GG
**FBG-C mutant 3**	Primer Forward	GTG GGA GAT GCC GCG ACT GCC TAC GCG CTG GCG GTG GAG GGG TAC
	Primer Reverse	GTA CCC CTC CAC CGC CAG CGC GTA GGC AGT CGC GGC ATC TCC CAC
**FBG-C mutant 4 and 5**	Primer Forward	AGC AAC TTC AGA AAT CTT GAA GGC GGG GGC TAA CGG GC
	Primer Reverse	GC CCG TTA GCC CCC GCC TTC AAG ATT TCT GAA GTT GC
**FBG-C mutant 6 and 7**	Primer Forward	GAC AAG GAC ACA CCT TCA GCC CTC ACC AGC TGT GCT CTG
	Primer Reverse	CAG AGC ACA GCT GGT GAG GGC TGA AGG TGT GTC CTT GTC
**FBG-X mutant 1**	Primer Forward	CAC GTA GAC TCG GCT AAG GCG AAG TAC CGC CTC CAC TTG
	Primer Reverse	CAA GTG GAG GCG GTA CTT CGC CTT AGC CGA GTC TAC GTG
**FBG-X mutant 2**	Primer Forward 1	GAC TCC TTC CAC GTA GGC GAT GCT AAG GCG AAG TAC CGC
	Primer Reverse 1	GCG GTA CTT CGC CTT AGC ATC GCC TAC GTG GAA GGA GTC
	Primer Forward 2	GTA GGC GAT GCT AAG ACG AAG TAC CGC CTC AAG TTG GAG GGC
	Primer Reverse 2	GCC CTC CAA CTT GAG GCG GTA CTT CGT CTT AGC ATC GCC TAC
**FBG-X mutant 3**	Primer Forward 1	TCC CCA GCG GGG AGA CGC AAA CTC GAG

		CAC CAC CAC
	Primer Reverse 1	GTG GTG GTG CTC GAG TTT GCG TCT CCC CGC TGG GG
	Primer Forward 2	CCA GCG GGG AGA CGC AAA CGC GCG TAG CAC CAC
	Primer Reverse 2	GTG GTG CTA GCG CGC TTT GCG TCT CCC CGC TGG
**FBG-X mutant 4**	Primer Forward	CGT GAT CGG GAC GAC AAC AGC TTG ATC ATC AAC TGC GCT GTC TCC
	Primer Reverse	GGA GAC AGC GCA GTT GAT GAT CAA GCT GTT GTC GTC CCG ATC ACG
